# Microbiome Landscape and Association with Response to Immune Checkpoint Inhibitors in Advanced Solid Tumors: A SCRUM-Japan MONSTAR-SCREEN Study

**DOI:** 10.1158/2767-9764.CRC-24-0543

**Published:** 2025-05-27

**Authors:** Kentaro Sawada, Riu Yamashita, Shunsuke A. Sakai, Satoshi Horasawa, Ayumu Yoshikawa, Takao Fujisawa, Shigenori Kadowaki, Ken Kato, Makoto Ueno, Eiji Oki, Yoshito Komatsu, Tatsuyuki Chiyoda, Yosuke Horita, Hisateru Yasui, Tadamichi Denda, Hironaga Satake, Taito Esaki, Taroh Satoh, Naoki Takahashi, Kentaro Yamazaki, Nobuhisa Matsuhashi, Tomohiro Nishina, Hiroyuki Takeda, Koushiro Ohtsubo, Takashi Ohta, Akihito Tsuji, Masahiro Goto, Takeshi Kato, Hideaki Bando, Katsuya Tsuchihara, Yoshiaki Nakamura, Takayuki Yoshino

**Affiliations:** 1 Department of Medical Oncology, Kushiro Rosai Hospital, Kushiro, Japan.; 2 Division of Translational Informatics, Exploratory Oncology Research and Clinical Trial Center, National Cancer Center, Kashiwa, Japan.; 3 Graduate School of Frontier Sciences, The University of Tokyo, Tokyo, Japan.; 4 Translational Research Support Office, National Cancer Center Hospital East, Kashiwa, Japan.; 5 Department of Gastrointestinal Oncology, National Cancer Center Hospital East, Kashiwa, Japan.; 6 Head and Neck Medical Oncology, National Cancer Center Hospital East, Kashiwa, Japan.; 7 Department of Clinical Oncology, Aichi Cancer Center Hospital, Nagoya, Japan.; 8 Department of Head and Neck Medical Oncology, National Cancer Center Hospital, Tokyo, Japan.; 9 Department of Gastroenterology, Kanagawa Cancer Center, Yokohama, Japan.; 10 Department of Surgery and Science, Kyushu University Hospital, Fukuoka, Japan.; 11 Department of Cancer Center, Hokkaido University Hospital, Sapporo, Japan.; 12 Department of Obstetrics and Gynecology, Keio University School of Medicine, Tokyo, Japan.; 13 Department of Medical Oncology, Saitama Medical University International Medical Center, Hidaka, Japan.; 14 Department of Medical Oncology, Kobe City Medical Center General Hospital, Kobe, Japan.; 15 Department of Gastroenterology, Chiba Cancer Center, Chiba, Japan.; 16 Cancer Treatment Center, Kansai Medical University Hospital, Hirakata, Japan.; 17 Department of Medical Oncology, Kochi Medical School, Nankoku, Japan.; 18 Department of Gastrointestinal and Medical Oncology, National Hospital Organization Kyushu Cancer Center, Fukuoka, Japan.; 19 Department of Frontier Science for Cancer and Chemotherapy, Graduate School of Medicine, Osaka University, Suita, Japan.; 20 Department of Gastroenterology, Saitama Cancer Center, Ina, Japan.; 21 Division of Gastrointestinal Oncology, Shizuoka Cancer Center, Nagaizumi, Japan.; 22 Department of Gastroenterological Surgery and Pediatric Surgery, Gifu University Graduate School of Medicine, Gifu, Japan.; 23 Gastrointestinal Medical Oncology, National Hospital Organization Shikoku Cancer Center, Gifu, Japan.; 24 Department of Clinical Oncology, St. Marianna University School of Medicine, Kawasaki, Japan.; 25 Division of Medical Oncology, Cancer Research Institute, Kanazawa University, Kanazawa, Japan.; 26 Department of Clinical Oncology, Kansai Rosai Hospital, Amagasaki, Japan.; 27 Department of Clinical Oncology, Kagawa University Hospital, Amagasaki, Japan.; 28 Cancer Chemotherapy Center, Osaka Medical and Pharmaceutical University Hospital, Takatsuki, Japan.; 29 Department of Surgery, National Hospital Organization Osaka National Hospital, Osaka, Japan.

## Abstract

Although the gut microbiome is associated with cancer development and progression, little is known about the effects of the gut microbiome landscape and the efficacy of immune checkpoint inhibitors (ICI) across cancer types. We investigated the association between the microbiome, clinical features, and ICI efficacy across cancer types in a large nationwide screening project for solid tumors. Among 2,180 patients with advanced solid tumors enrolled in the SCRUM-Japan MONSTAR-SCREEN between October 2019 and September 2021, in the chemotherapy-naïve cohort (*n* = 817), a high prevalence of oral bacteria was observed in patients using proton pump inhibitors (PPI) and those with upper gastrointestinal cancers, particularly postoperative patients with gastric or pancreatic cancer. Among patients treated with ICIs (*n* = 333), a high abundance of sequence variants in the gut microbiome was not significantly associated with ICI efficacy across cancer types (HR = 0.94; 95% confidence interval, 0.73–1.21). However, high oral bacteria in feces significantly correlated with a shorter progression-free survival compared with low oral bacteria (median, 4.34 vs. 6.97 months; HR = 1.38; 95% confidence interval, 1.07–1.78). Notably, in patients using PPIs, a higher proportion of oral bacteria influenced progression-free survival outcomes of ICI treatment (median, 3.15 vs. 2.04 months; *P* = 0.08), unlike in PPI nonusers (median, 7.13 vs. 5.55 months; *P* = 0.74). This study of the gut microbiome has unveiled significant insights into its landscape and potential impact on ICI efficacy. It highlights that the abundance of oral bacteria in feces may play a critical role in diminishing ICI efficacy among patients using PPIs.

**Significance:**

As part of the MONSTAR-SCREEN, a prospective nationwide project for patients with solid tumors, we found that although gut microbiome diversity does not consistently predict ICI efficacy across cancer types, a high level of oral bacteria in the gut is linked to reduced ICI effectiveness, especially in patients using PPIs. These findings highlight the potential clinical impact of microbiome variations on cancer treatment outcomes.

## Introduction

The gut microbiota strongly affect the development and progression of cancer by producing toxic metabolites and inducing inflammation or immunosuppression (1–3). Characteristic features of the gut microbiota in patients with cancer, such as an abundance of *Fusobacterium nucleatum* in individuals with colorectal cancer compared with healthy controls, are becoming increasingly evident. However, variations in the gut microbiota across different cancer types have been less extensively studied. Additionally, the immunomodulatory effects of the gut microbiota can affect not only prognosis but also the response or resistance to various therapeutic modalities, including immune checkpoint inhibitors (ICI; refs. 4–7). Despite substantial advances in ICI treatments for numerous cancer types, identifying tumor-derived biomarkers remains limited to a few factors, such as PD-L1 expression (8, 9), microsatellite instability (MSI; ref. 10), and tumor mutational burden (TMB; ref. 11). Consequently, there is a pressing need for more effective biomarkers to enhance ICI treatment response. Previous studies have linked high α diversity in the gut microbiota with a better response to ICIs in several cancer types (6, 7), and “favorite” and “unfavorite” bacterial species for ICI response were also reported (12, 13).

Moreover, recent studies have suggested that concomitant drugs, such as proton pump inhibitors (PPI) and probiotics, may alter ICI efficacy (14–16). Although it is thought that these effects are mediated by changes in the gut microbiome, it remains unclear whether there is a consistent association between gut microbiota and ICI response across various cancer types, as these studies involved cohorts with only one or a few types of cancer.

SCRUM-Japan MONSTAR-SCREEN, a nationwide advanced solid tumor biomarker screening project (17) was initiated to accelerate precision oncology. All enrolled patients underwent microbiome analysis of fecal samples and ctDNA analysis of blood samples. This study aims to explore the interactions between the gut microbiome and clinical characteristics, including cancer types, and to elucidate the association between the gut microbiome and ICI efficacy.

## Materials and Methods

### Study design and participants

**Figure 1:**
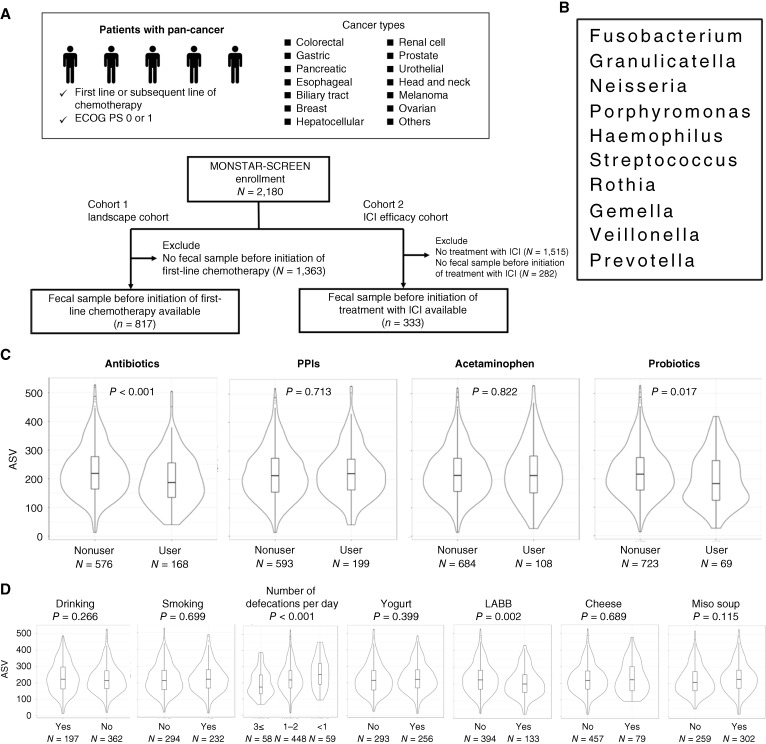
α diversity of gut microbiome according to clinical features and cancer types. **A,** Diagram depicting the selection of the MONSTAR-SCREEN cohorts. ECOG, Eastern Cooperative Oncology Group; PS, performance status. **B,** Definition of oral bacteria. A total of 10 genera were defined as oral bacteria in this study from the data of the article by Atarashi and colleagues. **C,** Violin plot of α diversity based on ASV counts in feces according to concomitant medication use. A two-sided *P* value was calculated with the Welch *t* test. **D,** Violin plot of α diversity by ASV count in feces according to lifestyle habit. A two-sided *P* value was calculated with the Welch *t* test. For the number of defecations per day, the Kruskal–Wallis test was applied. LABB, lactic acid bacteria beverage.

In this analysis of the 2,180 patients enrolled in the MONSTAR-SCREEN (17) between October 2019 and September 2021, cohort 1 included 817 patients with available fecal samples collected before initiating first-line systemic therapy (no modification of the microbiome due to cancer chemotherapy). These samples were analyzed for differences in the gut microbiome across various cancer types or lifestyles. Cohort 2 comprised 333 patients whose fecal specimens were obtained before treatment with ICIs, regardless of the treatment line, to assess the association between ICI efficacy and the gut microbiome (Fig. 1A). The project involved 31 core Japanese institutions. The main inclusion criteria for the SCRUM-Japan MONSTAR-SCREEN were (i) histopathologically confirmed unresectable or metastatic solid tumor other than lung cancer, (ii) receipt of or planned systemic therapy, (iii) age ≥16 years, (iv) an Eastern Cooperative Oncology Group performance status of 0 to 1, (v) adequate organ function, and (vi) life expectancy of ≥12 weeks. To avoid changes in the gut microbiome and suppression of ctDNA shedding because of chemotherapy, participants were required not to have started systemic chemotherapy or to be diagnosed with disease progression during systemic chemotherapy and not to have begun subsequent therapy at the time of fecal and blood sample collection. Written informed consent was obtained from all eligible patients. Fecal samples were collected using a commercial fecal sampling kit with a preservation solution (TechnoSuruga Laboratory Co., Ltd.) at each institution and sent to a central clinical laboratory for 16S rRNA amplicon sequencing. ctDNA genotyping was performed using FoundationOne Liquid CDx (Foundation Medicine). For untreated patients with available archival or fresh tumor tissue samples, additional genotyping tests were performed on these tissue samples using FoundationOne CDx (Foundation Medicine).

This study adhered to the Declaration of Helsinki and the Japanese Ethical Guidelines for Medical and Health Research Involving Human Subjects. The study protocol was approved by the Institutional Review Board of each participating institution and registered in the University Hospital Medical Information Network Clinical Trials Registry (protocol no. UMIN 000036749).

### Clinical data collection

Clinicopathological and efficacy data related to the systemic treatment of patients were collected using an electronic data capture system. The clinical data and genotyping results were stored in a clinical-grade database for integrated clinical genomic analysis. Concomitant medications, lifestyle habits, and dietary information were surveyed using patient questionnaires at the time of stool sample collection. For cohort 1, the definition of concomitant drug use (antibiotics, PPIs, probiotics, and acetaminophen) included usage within 3 months before treatment initiation. In cohort 2, the definition also encompassed usage during the ICI treatment period. Tumors were defined as MSI high if this status was detected using FoundationOne CDx or FoundationOne Liquid CDx. TMB was calculated using FoundationOne Liquid CDx, with TMB high defined as 10 mutations/mb or higher.

### Statistical assessment

This observational study did not perform sample size calculations based on formal statistical testing. Statistical comparisons were made using the χ^2^ or Fisher exact test for categorical data and the Mann–Whitney test for continuous data. Violin plots were generated using “ggplot2” in the R package (versions 3.3.6 or 3.4.0).

Progression-free survival (PFS) for ICI therapy was defined as the period from the date of ICI therapy initiation to the date of disease progression or death from any cause. Overall survival (OS) was defined as the period from the date of first-line therapy initiation to the date of death from any cause. Survival rates were estimated using the Kaplan–Meier method, and patient groups were compared using the log-rank test. HRs and 95% confidence intervals (CI) were calculated using the Cox proportional hazards model. Statistical analyses were performed using “ALDEx2” in the R package (versions 1.22.0 or 1.24.0) and IBM SPSS statistical software (version 28.0). The cutoff date for the data analysis was March 31, 2023. To adjust for patient backgrounds, propensity score matching was performed using cancer types in the OS analysis and both cancer types and treatment in the PFS analysis.

### Bacterial DNA extraction and 16S ribosome amplicon sequencing

The fecal samples were stored at room temperature for up to 7 days before being frozen at −80°C until DNA extraction. DNA was extracted from the fecal samples in a central laboratory following the manufacturer’s instructions. Briefly, a stool suspension (150 μL) in a preservative solution was used for DNA extraction using the NucleoSpin 96 Soil kit (Macherey-Nagel GmbH & Co. KG). The final volume of the extracted DNA was 100 μL, yielding approximately 50 μL of DNA solution. The extracted DNA was then purified using Agencourt AMPure XP beads (Beckman Coulter). DNA concentration was quantified using the PicoGreen dsDNA Assay Kit (Thermo Fisher Scientific).

For the first PCR, 1 ng of purified DNA served as the template using the 16S (V3–V4) Metagenomic Library Construction Kit for next-generation sequencing (Takara Bio). The PCR products were purified with Agencourt AMPure XP beads (Beckman Coulter). These purified products were then used as templates for index PCR with the Nextera XT Index Kit (Illumina). The indexed PCR products were further purified using Agencourt AMPure XP beads (Beckman Coulter) and quantified with the Quant-iT PicoGreen dsDNA Assay Kit (Thermo Fisher Scientific). An equimolar mixture of indexed PCR products, based on each sample's concentration, was used to prepare the library. The library’s size and concentration were determined using a TapeStation (Agilent Technologies). Sequencing was performed on a MiSeq sequencer using a 250 bp paired-end protocol with the MiSeq sequencing reagent kit v3 (Illumina), including approximately 40% to 50% of the PhiX Control (Illumina).

### Processing of 16S rRNA gene sequence reads

QIIME 2 v2022.2 was utilized for annotating FASTQ files (18). The reads were rarefied to 38,807, which represented the minimum number of reads across all samples. Low-quality sequences were filtered out, resulting in 48,277 amplicon sequence variants (ASV). Phylogenetic assignment was performed using the SILVA database (v138) to categorize the reads by taxonomy.

### Definition of oral bacteria in feces and their proportion

In this study, we identified 10 genera as oral bacteria (Fig. 1B), integrating 24 oral bacterial operational taxonomy units (OTU) provided by Atarashi and colleagues (19). These 24 OTUs are typically found in more than 1% of the salivary microbiota as demonstrated in Atarashi and colleagues’s work. We downloaded the bacteria list from https://www.science.org/doi/suppl/10.1126/science.aan4526/suppl_file/aan4526_atarashi_sm_tables_s1_to_s4.xlsx. The proportion of oral bacteria was calculated by summing the relative abundance of the taxonomies at the genus level corresponding to the list in our ASVs.

### Annotation of α or β diversity and Kyoto Encyclopedia of Genes and Genomes orthology

For α diversity analysis, our study utilized ASV counts, Shannon index (20), and Simpson index (21). Hierarchical clustering based on effect sizes by the ALDEx2 algorithm and the Ward method was employed to compare bacterial flora across different cancer types. β diversity was assessed using PERMANOVA to analyze variations between groups distinguished by concomitant medication, lifestyle habits, and cancer types. Furthermore, gene function estimation was performed using the PICRUSt2 v2.5.0 (22) tool, predicting Kyoto Encyclopedia of Genes and Genomes orthologs (KO) from 16S rRNA gene sequences. The quantities of ASVs and KO were defined as ASV and KO values, respectively.

### Data availability

The raw sequence data have been deposited with links to BioProject accession number PRJDB20380 in the DDBJ Sequence Read Archive. Further information and requests for resources and reagents should be directed to and will be fulfilled by the corresponding author.

## Results

### Lifestyle habits and bacterial flora in chemotherapy-naïve patients

Initially, we evaluated the impact of concomitant medication use and lifestyle habits on α and β diversities in chemotherapy-naïve patients. In this cohort (cohort 1), the median age was 68 years, with the most common cancer types being pancreatic (19.7%), colorectal (17.3%), biliary tract (7.8%), and gastric (6.6%). One hundred sixty-eight (20.6%) and 199 (24.4%) patients reported using antibiotics and PPIs, respectively, within 3 months prior to treatment initiation. Additional patient information, including lifestyle and dietary habits, is presented in Table 1.

**Table 1:** Patient characteristics in cohort 1

Characteristic	Total (*n* = 817) *N* (%)
Age	
Median (range)	68 (22–88)
Sex	
Male	453 (55.4)
Female	364 (44.6)
Cancer type	
Pancreatic	161 (19.7)
Colorectal	141 (17.3)
Biliary tract	64 (7.8)
Gastric	54 (6.6)
Head and neck	54 (6.6)
Breast	47 (5.8)
Urothelial	47 (5.8)
Renal cell	47 (5.8)
Ovary	45 (5.5)
Malignant melanoma	41 (5.0)
Prostate	41 (5.0)
Hepatocellular	20 (2.4)
Endometrial	19 (2.3)
Neuroendocrine neoplasm	17 (2.1)
Cervical	7 (0.9)
Esophageal	6 (0.7)
Small intestine	2 (0.2)
GIST	2 (0.2)
Merkel cell	2 (0.2)
Antibiotic use	
Yes	168 (20.6)
No	576 (70.5)
Unknown	73 (8.9)
PPI use	
Yes	199 (24.4)
No	593 (72.6)
Unknown	25 (3.1)
Acetaminophen use	
Yes	108 (13.2)
No	684 (83.7)
Unknown	25 (3.1)
Probiotics use	
Yes	69 (8.4)
No	723 (88.5)
Unknown	25 (3.1)
Alcohol drinking	
Yes	197 (24.1)
No	362 (44.3)
Unknown^a^	258 (31.6)
Smoking	
Yes	232 (28.4)
No	294 (36.0)
Unknown^a^	291 (35.6)
Number of defecations per day	
3≤	58 (7.1)
1–2	448 (54.8)
<1	59 (7.2)
Unknown^a^	252 (30.8)
Habitual intake of yogurt	
Yes (four or more times a week)	256 (31.3)
No (three or less times a week)	293 (35.9)
Unknown^a^	268 (32.8)
Habitual intake of LABB	
Yes (four or more times a week)	133 (16.3)
No (three or less times a week)	394 (48.2)
Unknown^a^	290 (32.8)
Habitual intake of cheese	
Yes (four or more times a week)	79 (9.7)
No (three or less times a week)	457 (55.9)
Unknown^a^	281 (34.4)
Habitual intake of miso soup	
Yes (four or more times a week)	302 (37.0)
No (three or less times a week)	259 (31.7)
Unknown^a^	256 (31.3)

Abbreviations: GIST, GI stromal tumor; LABB, lactic acid bacteria beverage.

a In the life habit (from alcohol to habitual intake of miso soup), “Unknown” included patients who answered “Yes” in the use of antibiotics in order to exclude the effects of antibiotic-induced dysbiosis.

In patients whose microbiome was not modified by cancer chemotherapy, those with concomitant antibiotic or probiotic use—primarily prescribed medications—exhibited significantly lower ASV counts and Shannon indices compared with those not using these medications (*P* < 0.001 and *P* = 0.017 for ASVs and *P* < 0.001 and *P* = 0.021 for Shannon, respectively; Fig. 1C; Supplementary Fig. S1A). The composition of bacterial flora also differed significantly according to PERMANOVA analysis (*P* < 0.001 for antibiotics and *P* = 0.003 for probiotics; Supplementary Fig. S2A). In the ALDEx2 analysis, *Enterococcus, Enterobacteriaceae*, and *Veillonella* were statistically significantly more abundant in the feces of patients using antibiotics (Supplementary Fig. S2B). Patients using PPIs showed a significantly different flora composition (PERMANOVA analysis, *P* < 0.001) and tended to have lower α diversity as measured by the Simpson index (*P* = 0.072) although other α diversity indices such as ASVs and the Shannon index did not significantly differ from patients not using PPIs (*P* = 0.713 and *P* = 0.651, respectively; Fig. 1C; Supplementary Fig. S1A and S2A). Interestingly, in the subset of patients not using antibiotics, frequent consumption (defined as four or more times a week) of a lactic acid bacteria beverage and frequent defecations (defined as three or more times per day) were associated with lower ASV counts (*P* = 0.002 and *P* < 0.001, respectively) and Shannon indices (*P* = 0.009 and *P* = 0.013, respectively; Fig. 1D; Supplementary Fig. S1B). These factors also correlated with different flora compositions according to PERMANOVA analysis (*P* = 0.012 and *P* < 0.001, respectively; Supplementary Fig. S2C). Yogurt and miso soup intake were linked to alterations in flora composition (*P* = 0.035 and *P* = 0.024, respectively) but did not affect individual α diversity indices. Other lifestyle habits, such as smoking and alcohol drinking, were not statistically associated with α or β diversity.

### Cancer types and gut microbiome composition

**Figure 2:**
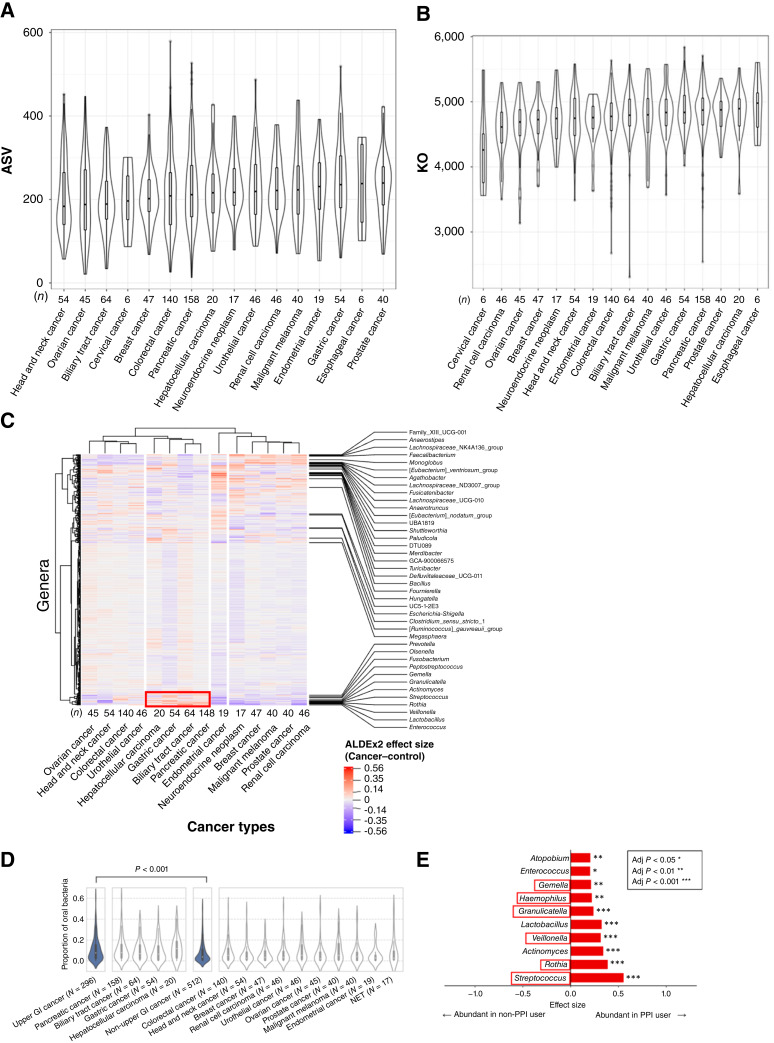
Details of microbiome according to cancer types and PPI use in cohort 1. **A,** α diversity based on the observed number of ASVs according to cancer type, which was based on the analysis of at least five specimens obtained from patients in cohort 1 (*n* = 817). **B,** α diversity based on the observed number of KO indices according to cancer type, which was based on the analysis of at least five specimens obtained from patients in cohort 1 (*n* = 817). **C,** Heatmaps depicting relative increases and decreases in bacterial species in patients with each cancer type compared with all patients as measured using 16S rRNA analysis and presented as ALDEx2 effect size. The distance between cancer types was used to determine effect sizes using the ALDEx2 algorithm, and hierarchical clustering was performed using the Ward method. Red and blue indicate bacterial species that were abundant or scarce, respectively, compared with the reference (all samples). The area boxed in red indicates increased levels of oral bacteria in patients with upper GI cancer. Only the genera with the top 1% of the largest absolute effect size are listed on the right-hand side of the figure. **D,** Violin plot of the proportion of oral bacteria in the feces of patients with upper GI cancer (*n* = 296) vs. those with non-upper GI cancer (*n* = 512). NET, neuroendocrine tumor. **E,** Differences in fecal bacterial flora between PPI users (*n* = 199) and non-users (*n* = 593) based on ALDEx2. An adjusted *P* value < 0.05 and an absolute value of effect size >0.2 were used to define abundant species. Species defined as oral bacteria are indicated by red boxes.

To clarify the differences in α diversity based on cancer type, we compared α and β diversities among 16 distinct cancer types, each with fecal samples from at least five patients. The median ASV counts did not significantly differ among patients with different cancer types (one-way ANOVA, *P* = 0.155), ranging from 183.5 in patients with head and neck cancer to 239.5 in those with prostate cancer (Fig. 2A). However, the median Shannon and Simpson indices differed among cancer types (one-way ANOVA; *P* = 0.006 and *P* = 0.002, respectively; Supplementary Fig. S3A and S3B), and β diversity was significantly different between cancer types (*P* < 0.001). To examine functional diversity, we analyzed functional orthologs using the KO database. The median number of KO identifiers significantly differed across cancer types (one-way ANOVA, *P* = 0.003), ranging from 4,260 in patients with cervical cancer to 4,979 in patients with esophageal cancer (Fig. 2B).

For a more detailed comparison of bacterial flora beyond α diversity, we evaluated the composition of bacterial flora across patients with different cancer types using clustering analysis. We excluded cancer types with fewer than 10 available fecal samples for the clustering analysis. In total, we obtained 790 fecal microbiome samples from 14 cancer types. The results of the clustering analysis revealed that all cancer types could be classified into four major groups, “clusters”, with similar gut microbiome composition. Notably, cancers of anatomically proximal organs such as hepatocellular carcinoma, gastric cancer, biliary tract cancer, and pancreatic cancer were grouped together in the upper gastrointestinal (GI) cancer group according to their gut microbiome compositions (Fig. 2C). When oral bacteria were defined as the 10 genera listed in Fig. 1B, comprising 24 OTUs typically present at more than 1% in the salivary microbiota as reported by Atarashi and colleagues (19), hierarchical clustering showed that specific oral bacteria were enriched in upper GI cancers (Fig. 2C). To confirm the enrichment of oral bacteria in patients with upper GI cancer, we compared the proportion of total oral bacteria between patients with upper GI cancer (*n* = 296) and those with other cancers (*n* = 512). The proportion of oral bacteria was significantly higher in patients with upper GI cancer than in those with other cancers (median, 7.7% vs. 3.8%; *P* < 0.001; Fig. 2D).

To evaluate the influence of surgical intervention on the enrichment of oral microbes in feces from patients with upper GI cancer, we compared the gut microbiome of patients with or without primary tumor resection within this group using ALDEx2 analysis. Primary tumor resection in patients with gastric cancer and pancreatic cancer was associated with a significantly greater abundance of oral bacteria in feces (*P* = 0.010 and *P* = 0.031, respectively) although no significant associations were found among patients with hepatocellular carcinoma and biliary tract cancer (Supplementary Fig. S4). Furthermore, to explore the relationship between gastric acid exposure and oral bacteria in feces, we examined the influence of concomitant PPIs, potent gastric acid suppressants, on the presence of oral bacteria in the feces of patients in cohort 1. Concomitant PPI use was defined as use within 3 months prior to initiating systemic chemotherapy. The detailed cancer types of PPI users (*n* = 199) and nonusers (*n* = 593) are shown in Supplementary Table S1, with PPIs being more frequently used by patients with upper GI cancer than those with non-GI cancer (Fisher exact test, *P* < 0.001). We found that 10 genera were significantly abundant in the fecal samples of PPI users, and six of these were classified as oral bacteria (e.g., *Streptococcus*, *Rothia*, and *Veillonella*; Fig. 2E). In contrast, no genera were particularly abundant among PPI nonusers.

### Association between bacterial diversity and the efficacy of ICIs

Next, we evaluated the association between the fecal microbiome and ICI efficacy using fecal samples from patients treated with ICIs (cohort 2). In cohort 2, the most prevalent cancer types were head and neck cancer (17.4%), renal cell cancer (15.6%), malignant melanoma (13.8%), and urothelial cancer (13.5%). Notably, about half of the patients (47.4%) received ICIs as a first-line treatment. Of these, 267 (80.2%) were treated with ICIs alone, 36 (10.8%) with ICIs plus a molecularly targeted agent, and 30 (9.0%) with ICIs plus chemotherapy. MSI-high and TMB-high statuses were detected in 12 (3.6%) and 68 (20.4%) patients, respectively (Table 2). Details of concomitant medication use, including antibiotics and PPIs, sorted by cancer type, are provided in Supplementary Table S2.

**Table 2:** Patient characteristics in cohort 2

Characteristic	Total (*n* = 333) *N* (%)
Age	
Median (range)	69 (19–88)
Sex	
Male	214 (64.3)
Female	119 (35.7)
Cancer type	
Head and neck	58 (17.4)
Renal cell	52 (15.6)
Malignant melanoma	46 (13.8)
Urothelial	45 (13.5)
Gastric	41 (12.3)
Esophageal	36 (10.8)
Hepatocellular	16 (4.8)
Breast	15 (4.5)
Colorectal	6 (1.8)
Prostate	5 (1.5)
Endometrial	3 (0.9)
Pancreatic	3 (0.9)
Merkel cell	2 (0.6)
Small intestine	2 (0.6)
Biliary tract	1 (0.3)
Ovary	1 (0.3)
Cervical	1 (0.3)
Treatment line	
1st	158 (47.4)
2nd or later	175 (52.6)
Treatment	
ICIs alone	267 (80.2)
Anti–PD-1 or anti–PD-L1	223 (67.0)
Anti–PD-1+ anti–CTLA-4	44 (13.2)
ICIs + molecular target agent	36 (10.8)
ICIs + chemotherapy	30 (9.0)
MSI status (tissue or liquid)	
High	12 (3.6)
Low	321 (96.4)
TMB status (high; ≥10 mut/Mb with tissue or liquid)	
High	68 (20.4)
Low	265 (79.6)

Abbreviation: mut, mutations.

**Figure 3:**
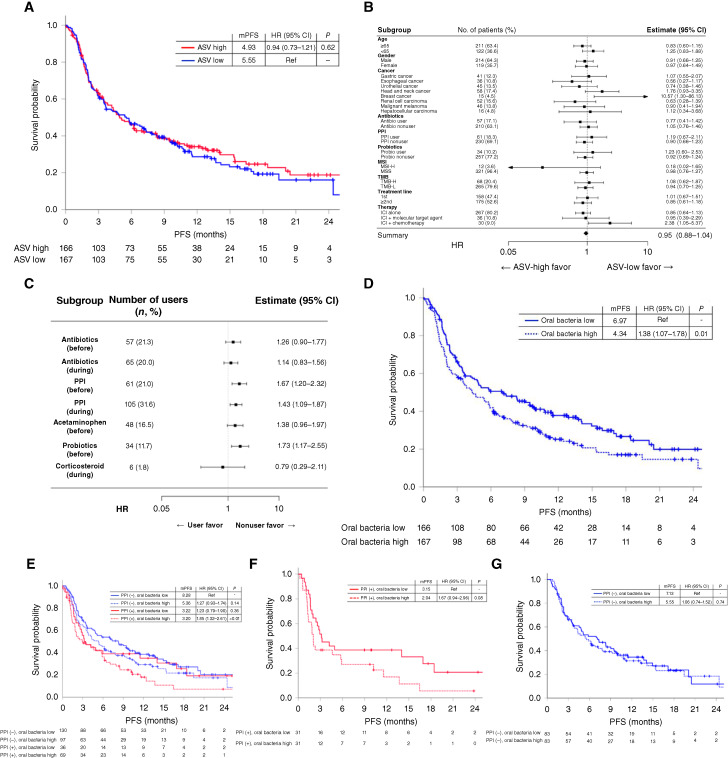
Relationship between ICI efficacy and bacterial flora/concomitant medication use. **A,** Kaplan–Meier plots of the PFS of patients treated with ICIs in the high ASV group and the low ASV group. The ASV cutoff was 206.0 (median). **B,** Forest plot analysis depicting the association between clinical features and PFS in patients with ICIs. Dots and error bars represent point estimates of the HR and 95% CIs. Antibio, antibiotics; MSI-H, MSI high; MSS, microsatellite stable; TMB-H, TMB high; TMB-L, TMB low; Probio, probiotics. **C,** Forest plot of the impact of concomitant medication use on the PFS of patients treated with ICIs. “Before” was defined as use within 3 months before the initiation of ICI treatment. “During” was defined as use during ICI treatment. **D,** Kaplan–Meier plots of PFS of patients treated with ICIs based on the proportion of oral bacteria in feces. “Oral bacteria high” was defined as patients with the proportion of oral bacteria above median. **E,** Kaplan–Meier plots of the PFS of patients treated with ICIs based on PPI use and the abundance of oral bacteria in the feces. “Oral bacteria high” was defined as patients with the proportion of oral bacteria above median. **F,** Kaplan–Meier plots of the PFS according to the proportion of oral bacteria in patients with PPI use after propensity score matching. **G,** Kaplan–Meier plots of the PFS according to the proportion of oral bacteria in patients without PPI use after propensity score matching. mPFS, median PFS; Ref, reference.

Initially, we explored the correlation between α diversity and PFS with ICI therapy. Based on the median ASV count of 206.0, both the high ASV group (*n* = 166) and the low ASV group (*n* = 167) demonstrated similar PFS (median, 4.93 vs. 5.55 months; HR = 0.94; 95% CI, 0.73–1.21; Fig. 3A). Additionally, the high Shannon group (*n* = 166) showed no association with improved ICI efficacy (median, 5.88 vs. 4.76 months; HR = 0.84; 95% CI, 0.65–1.10; Supplementary Fig. S5A). However, the high Simpson index group was associated with favorable ICI efficacy (median, 6.77 vs. 4.21 months; HR = 0.76; 95% CI, 0.59–0.97; Supplementary Fig. S5B).

Subgroup analysis of PFS and ASV counts revealed similar ICI efficacy between the ASV-high and ASV-low groups, irrespective of age, sex, antibiotic, PPI or probiotic use, MSI status, TMB status, or treatment line (Fig. 3B). Although no significant difference in PFS was noted between the ASV-high and ASV-low groups overall, subgroup analysis by cancer type indicated that patients with esophageal, urothelial, and renal cell carcinoma in the ASV-high group tended to have longer PFS (HR = 0.56, 0.74, and 0.63, respectively). Conversely, patients with head and neck cancer and breast cancer exhibited shorter PFS (HR = 1.76 and 10.57, respectively) although these findings were exploratory and mostly not statistically significant. Patients treated with ICIs alone (*n* = 267) showed a trend toward longer PFS in the high ASV group compared with the low ASV group (median, 4.53 vs. 3.65 months; HR = 0.85; 95% CI, 0.65–1.13; Supplementary Fig. S6A) although this difference was not statistically significant. For patients receiving ICIs plus molecularly targeted agents (*n* = 36), PFS was similar between both groups (median, 12.42 vs. 9.03 months; HR = 0.95; 95% CI, 0.39–2.29; Supplementary Fig. S6B). In contrast, those on ICIs plus chemotherapy (*n* = 30) exhibited longer PFS in the ASV-low group compared with the high ASV group (median, 3.45 vs. 11.50 months; HR = 2.38; 95% CI, 1.05–5.38; Supplementary Fig. S6C) although these analyses involved small sample sizes. Additionally, further microbiome analysis based on PFS of patients receiving ICIs revealed that certain oral bacteria like *Streptococcus*, *Gemella*, and *Rothia* were prevalent in the short PFS group, whereas *Blautia* and *Faecalibacterium*, previously reported as “favorite” bacteria in prior studies (5, 23), were abundant in the long PFS group (Supplementary Fig. S7).

### Association between concomitant drugs and/or oral bacteria and the efficacy of ICIs

Because concomitant medication use affected α diversity and the proportion of oral bacteria in feces in cohort 1, we investigated the effects of concomitant medication use and/or oral bacteria on ICI efficacy in cohort 2. Although the use of antibiotics before or during ICI treatment did not significantly affect ICI efficacy (HR = 1.26; 95% CI, 0.90–1.77 and HR = 1.14; 95% CI, 0.83–1.56), the use of PPIs and probiotics before ICI treatment significantly reduced efficacy [HR = 1.67 (95% CI, 1.20–2.32) for PPIs, HR = 1.43 (95% CI, 1.09–1.87) for probiotics, and HR = 1.73 (95% CI, 1.17–2.55) for probiotics before ICI treatment, respectively; Fig. 3C]. Even after adjusting for cancer types and treatment modalities (ICI monotherapy or ICIs plus other agents) using propensity score matching, the use of PPIs before or during ICI treatment tended to be associated with longer PFS compared with nonusers (*P* = 0.09 for both comparisons). However, probiotic use was not associated with ICI efficacy (*P* = 0.30; Supplementary Fig. S8A–S8C). In the ALDEx2 analysis of concomitant medication use in cohort 2, certain oral bacteria, including *Streptococcus*, *Rothia*, and *Granulicatella*, were enriched in patients who received PPIs before ICI treatment, mirroring the results obtained in cohort 1. However, no other drugs were associated with an enrichment of oral bacteria (Supplementary Fig. S9A–S9D).

Given the association between PPI use and reduced ICI efficacy, along with increased fecal levels of oral bacteria in PPI users, we next examined the relationship between fecal oral bacteria and ICI efficacy. Patients in cohort 2 were divided into four groups based on the proportion of oral bacteria in their feces. The median PFS for the top 25%, 25% to 50%, 50% to 75%, and bottom 25% were 3.84, 4.67, 5.82, and 7.36 months, respectively (Supplementary Fig. S10A). A trend of decreasing median PFS with increasing proportions of fecal oral bacteria was observed. Patients were then categorized into two groups based on the median relative proportion of fecal oral bacteria (characteristics of each group are shown in Supplementary Table S3). The group with a high proportion of oral bacteria demonstrated a significantly shorter PFS compared with the low group (median, 4.34 vs. 6.97 months; HR = 1.38; 95% CI, 1.07–1.78; Fig. 3D). This difference persisted after adjusting for cancer type and treatment using propensity score matching (median, 4.63 vs. 5.98 months; HR = 1.35; 95% CI, 1.03–1.76; Supplementary Fig. S10B). In multivariate analysis, a high proportion of oral bacteria was the second strongest predictive factor of ICI efficacy HR = 1.31; 95% CI, 1.00–1.71; *P* = 0.048) following cancer types (upper GI cancer or others; HR = 2.26; 95% CI, 1.71–2.99; *P* < 0.001; Supplementary Table S4).

To investigate whether oral bacteria might be prognostic for OS rather than predictive of ICI efficacy, we evaluated the impact of fecal oral bacteria proportions on survival prognosis using data from cohort 1. After adjusting for cancer type using propensity score matching, the proportion of oral bacteria in feces was not associated with survival prognosis (median: high oral bacteria group 27.43 months vs. low oral bacteria group 27.20 months; HR = 1.03; 95% CI, 0.83–1.27; *P* = 0.79; Supplementary Fig. S10C).

Given that both PPI use and increased fecal levels of oral bacteria were associated with reduced ICI efficacy, we next aimed to ascertain the impact of the interaction between PPIs and oral bacteria on ICI efficacy. We combined data on PPI use and the proportions of oral bacteria and then analyzed PFS in cohort 2. Patients were divided into four categories based on PPI use and the relative proportion of oral bacteria (above or below median) in feces (patient characteristics are shown in Table 3). The data revealed that ICI efficacy was the lowest in patients who used PPIs and had a high proportion of oral bacteria in their feces (median PFS, 3.20 months). Conversely, patients who did not use PPIs and had a low proportion of oral bacteria in their feces had the longest PFS (median, 8.28 months; Fig. 3E). Interestingly, among PPI users, those with a low proportion of fecal oral bacteria tended to have longer PFS than those with a high proportion of oral bacteria after adjusting for cancer type and treatment by propensity score matching (median, 3.15 vs. 2.04 months; *P* = 0.08; Fig. 3F). In contrast, among non-PPI users, the proportion of oral bacteria was not associated with ICI efficacy (median, 7.13 vs. 5.55 months; *P* = 0.74; Fig. 3G).

**Table 3:** Patient characteristics of four groups based on PPI use and the abundance of oral bacteria in the feces

Characteristic	PPI (+)	PPI (+)	PPI (−)	PPI (−)
	Oral high (*n* = 69)	Oral low (*n* = 36)	Oral high (*n* = 97)	Oral low (*n* = 130)
	*N* (%)	*N* (%)	*N* (%)	*N* (%)
Age				
Median (range)	70 (42–84)	71 (19–83)	70 (32–88)	68 (21–83)
Sex				
Male	44 (63.8)	29 (80.6)	69 (71.1)	72 (55.4)
Female	25 (36.2)	7 (19.4)	28 (38.9)	58 (44.6)
Cancer type				
Head and neck	7 (10.1)	6 (16.7)	16 (16.5)	29 (22.3)
Renal cell	11 (15.9)	6 (16.7)	9 (9.3)	26 (20.0)
Malignant melanoma	5 (7.2)	6 (16.7)	13 (13.4)	22 (16.9)
Urothelial	11 (15.9)	2 (5.6)	15 (15.5)	17 (13.1)
Gastric	15 (21.7)	4 (11.1)	13 (13.4)	9 (6.9)
Esophageal	10 (14.5)	6 (16.7)	16 (16.5)	4 (3.1)
Hepatocellular	4 (5.8)	3 (8.3)	6 (6.2)	3 (2.3)
Breast	0	1 (2.8)	3 (3.1)	11 (8.5)
Colorectal	3 (4.3)	0	1 (1.0)	2 (1.5)
Prostate	1 (1.4)	0	2 (2.1)	2 (1.5)
Endometrial	0	1 (2.8)	0	2 (1.5)
Pancreatic	2 (2.9)	0	1 (1.0)	0
Merkel cell	0	0	1 (1.0)	1 (0.8)
Small intestine	0	1 (2.8)	1 (1.0)	0
Biliary tract	0	0	0	1 (0.8)
Ovary	0	0	0	1 (0.8)
Tumor biomarker				
MSI high	3 (4.3)	1 (3.0)	2 (2.1)	6 (4.6)
TMB high	13 (18.8)	9 (27.3)	19 (19.6)	26 (20.0)

Furthermore, we conducted MetaCyc pathway analysis to determine which microbial pathways were affected in patients with a higher presence of oral bacteria. In both cohorts 1 and 2, pathways related to *Staphylococcus aureus* infection, endocytosis, and bacterial invasion of epithelial cells were upregulated in patients with a higher presence of oral bacteria (cutoff: median; Supplementary Fig. S10D).

### Validation of the impact of oral bacteria on the efficacy of ICIs using public data

Next, we evaluated the association between the proportion of oral bacteria and ICI efficacy using publicly available data from 39 patients with malignant melanoma treated with ICIs (7). As previously mentioned, our study defined oral bacteria using 24 OTUs typically present at >1% in the salivary microbiota as described by Atarashi and colleagues (19). In the public dataset, seven of these 24 OTUs were evaluable. Comparing the microbiome profiles of responders and nonresponders, we found that certain oral bacteria such as *Rothia mucilaginosa* and *Streptococcus oralis* tended to be more abundant in ICI nonresponders (*P* = 0.11 and 0.13, respectively). The combined *P* value for these seven oral bacteria was 0.28 (Supplementary Fig. S11).

## Discussion

To elucidate the interaction between the microbiome and clinical characteristics, including the efficacy of ICIs, in patients with advanced solid tumors, we conducted an exploratory microbiome analysis using fecal samples from patients enrolled in MONSTAR-SCREEN. This large-scale, cross-organ cohort study analyzed the gut microbiome of more than 800 patients with advanced solid tumors, demonstrating variations in both α and β diversities among different cancer types using several indices. Notably, patients with upper GI cancers exhibited a distinct microbiome profile characterized by the enrichment of common oral bacteria. Furthermore, the microbiome composition was influenced by patient characteristics such as lifestyle and concomitant medication use, even within the same cancer type. Our findings suggest that an increase in fecal oral bacteria, in conjunction with PPI use, may decrease the efficacy of ICIs. To our knowledge, this is the first study to explore the relationship between the gut microbiome and clinical characteristics across cancer types, including an exploratory analysis of the association between PPI use and oral bacteria proportion with ICI efficacy.

Changes in the GI environment, such as reduced gastric acid exposure as a result of PPI use and/or gastrectomy, seem to influence the presence of oral bacteria in feces. Our results support previous studies that demonstrated an association between PPI use and an increase in oral bacteria in feces (24, 25) and the findings of a recent randomized trial showing that PPI use promotes the translocation of oral bacteria into the intestines (26). It is noteworthy that concordant results were observed in a Japanese cohort, regardless of the patients’ diet, genetics, and geographic location.

This study found an increased proportion of oral bacteria in the fecal microbiome of patients with upper GI cancers, particularly those with gastric or pancreatic cancer who had undergone primary tumor resection. Previous studies have shown that oral bacteria such as *Veillonella* and *Lactobacillus* are abundant in fecal samples from patients with gastric and pancreatic cancers (27–29). Notably, this is the first study to demonstrate further enrichment of oral bacteria following primary tumor resection. Conversely, our analysis did not show the enrichment of oral bacteria in colorectal cancer, which has been well documented in multiple independent studies (30–32). This discrepancy may be attributed to the unique background of our study, in which the control group comprised patients with solid tumors rather than healthy individuals. Additionally, our analysis methods—hierarchical clustering and the Ward method, which consider overall bacterial flora composition similarity between cancer types rather than focusing solely on oral bacteria—may contribute to this discrepancy.

Our study demonstrates that α diversity in the microbiome is not a consistent biomarker for ICI efficacy across cancer types. Although the Simpson index was associated with efficacy, the ASV count and Shannon index were not. This finding contrasts with previous reports encompassing several cancer types (6, 7), which found high α diversity to be associated with better ICI efficacy. This discrepancy may be attributed to varying predictive values across cancer types. In subgroup analysis, high ASV counts were associated with favorable ICI efficacy in esophageal, urothelial, and renal cell cancers, but with unfavorable ICI efficacy in head and neck cancer and breast cancer, although these findings are from exploratory analysis and most of these differences were not statistically significant. These results do not negate the theory that fecal α diversity predicts ICI treatment response but suggest that its predictive value may depend on the cancer type. Another possible explanation is the inclusion of patients treated with ICI monotherapy and ICIs plus other agents in this study. Although the sample size was small, patients treated with ICIs plus chemotherapy (*n* = 30) tended to show lower ICI efficacy in the high ASV group, whereas patients treated with ICI monotherapy (*n* = 267) tended to show slightly higher efficacy. These results suggest that although a “favorable” biome with high α diversity may activate antitumor immunity and enhance the effect of ICI monotherapy, the efficacy of ICIs combined with chemotherapy may be relatively unaffected by the biome because of the predominant impact of chemotherapy-induced cytotoxicity. ICIs combined with chemotherapy regimens have recently been approved as standard treatments for many cancer types, including gastric, esophageal, and head and neck cancers. Our results suggest that such treatment regimens may overcome ICI resistance stemming from an “unfavorable” microbiome, such as low α diversity, in certain cancer types.

With regard to specific bacteria related to the ICI efficacy, Hakozaki and colleagues (33) have shown that *Ruminococcaceae* and *Agathobacter* were more abundant in the feces of responders than nonresponders in the cohort of Japanese patients with non–small cell lung cancer, based on 16S RNA analysis. In our cohort, *Agathobacter* was also more abundant in patients with longer PFS of ICI, and it suggested that *Agathobacter* may be a potential predictor of ICI response across cancer types. Furthermore, *Ruminococcaceae* was less abundant in patients using PPIs in our cohort. Although previous studies have shown decreased ICI efficacy in PPI users, the mechanism of this reduction was unclear as the molecular mechanism of PPIs (covalently binding to the H^+^/K^+^-ATPase antiporter pumps of gastric parietal cells) has not been reported to directly inhibit the molecular activity of ICI agents. Meanwhile, PPI use has been shown to decrease the relative abundances of “favorable” bacteria such as *Ruminococcaceae* (as also shown in our study) and *Faecalibacterium spp.* (4, 34) leading to the hypothesis that PPI-induced dysbiosis may be related to reduced ICI efficacy. Moreover, our exploratory data, showing an increase in oral bacteria due to PPI use and subsequent reduction in ICI efficacy, support this hypothesis and provide important insights into the mechanism of PPI-induced reduction of ICI efficacy. In the validation study using public data to examine the relationship between oral bacterial abundance and ICI efficacy, two oral bacteria, *Rothia mucilaginosa* and *Streptococcus oralis*, tended to be abundant in ICI nonresponders. Although the sum of seven oral bacteria extracted from a public database did not show a trend toward higher abundance in nonresponders statistically (*P* = 0.28), possibly because of the small sample size, this result supported our data on the association between oral bacterial abundance and ICI efficacy. Interestingly, in our study, the impact of oral bacterial proportion in fecal samples on ICI efficacy was more pronounced in patients using PPIs compared with those not using PPIs. This suggests that for patients requiring PPI use during ICI treatment, such as for reducing gastric mucosal damage, implementing oral care to reduce the influx of oral bacteria into the intestines may potentially prevent the decrease in ICI efficacy. In contrast, this study did not show a statistically significant reduction in the efficacy of ICIs because of antibiotic use despite the results of previous large-scale studies. However, the HRs of PFS in cases of antibiotic use before and during ICI treatment were greater than one. The discrepancy may be simply caused by the small number of antibiotic use cases in this study. In any case, indiscriminate antibiotic use in ICI treatment should be avoided.

There are several limitations to this study. The first limitation is using only 16S rRNA sequencing data to assess the microbiome. The limitation of 16S rRNA sequencing includes low reproducibility due to short read lengths and variation in results depending on the selected region (35–37). Ideally, metagenomic sequencing would have been performed on all cases; however, it was not feasible primarily because of financial constraints in planning this large cohort study. Nonetheless, 16S rRNA sequencing remains a useful analysis method as previous reports have shown that it yielded similar results to shotgun metagenomic sequencing in terms of α diversity and prediction accuracy in patients with ulcerative colitis (38), and recent skin microbiome analysis in patients with breast cancer using 16S rRNA sequencing has provided novel insights (39). Nevertheless, employing additional detailed analysis methods, such as shotgun metagenomics (40, 41) or single-cell analysis (42), may allow for a much deeper characterization of microbiome complexity and identify more detailed microbiomes that predict ICI treatment efficacy. Second, patients treated with various treatment lines and regimens were included in the ICI efficacy analysis. The inclusion of patients who received various ICIs across different treatment lines may have complicated the evaluation of absolute PFS values in this study. However, the associations between α diversity and ICI efficacy were similar among patients who underwent first-, second-, or later-line treatments. This suggests that the bias introduced by including multiple treatment lines was not a major issue. Furthermore, including patients treated with various ICI regimens allowed us to examine the relationship between the microbiome and ICI efficacy according to specific treatment regimens. In the future, investigating interventions for the gut microbiome with prebiotics or fecal microbiota transplantation in patients treated with ICIs alone, which was shown to be particularly associated with the α diversity of the gut microbiome in this study, may improve ICI efficacy. Third, this study was not based on a strictly pre-planned statistical analysis but rather an exploratory analysis. Additionally, many of the HRs have confidence intervals that overlap one. Therefore, it is necessary to verify our results in different cohorts.

In conclusion, this is the first study to investigate the relationship between gut microbiome features and the efficacy of ICIs across different cancer types. We demonstrate an association between the gut microbiome and the mechanisms of PPI-induced attenuation of ICI efficacy. These exploratory findings may provide important insights for future studies aimed at overcoming ICI resistance in different cancer types.

## Supplementary Material

Supplementary Figure S1 The association with concomitant drugs / lifestyle habits and alpha diversity with Shannon and Simpson index.

Supplementary Figure S2 The association with concomitant drugs / lifestyle habits and beta diversity.

Supplementary Figure S3 The association with cancer types and alpha diversity with Shannon and Simpson index.

Supplementary Figure S4 Proportion of oral bacteria in patients with upper gastrointestinal cancers feces according to the presence or absence of a primary tumor by cancer type.

Supplementary Figure S5 Association of ICI efficacy with ASV based on treatment.

Supplementary Figure S6 Association of ICI efficacy with ASV based on treatment.

Supplementary Figure S7 The ALDEx2 analysis of flora according to PFS of ICI treatment.

Supplementary Figure S8 Association of ICI efficacy with concomitant medication use.

Supplementary Figure S9 ALDEx2 analysis of the comparison of the fecal microbiome in patients with or without concomitant medication use before ICI treatment.

Supplementary Figure S10 ICI efficacy between two categories of the proportion of oral bacteria in feces.

Supplementary Figure S11 Validation with publicly data about oral bacteria and the efficacy of ICIs

Supplementary Table S1 Patient characteristics of PPI user and non-user in Cohort 1.

Supplementary Table S2 Concomitant drug usage in each cancer type in Cohort 2.

Supplementary Table S3 Patient characteristics of two groups based on the proportion of oral bacteria in feces in cohort 2.

Supplementary Table S4 Multivariate analysis in PFS of ICIs in cohort 2.
