# Supplementary material for: Microbiome Landscape and Association with Response to Immune Checkpoint Inhibitors in Advanced Solid Tumors: A SCRUM-Japan MONSTAR-SCREEN Study
Source: Cancer Res Commun. 2025 May 27;5(5):857–70. doi: 10.1158/2767-9764.CRC-24-0543 (PMC12107420; doi:10.1158/2767-9764.CRC-24-0543)
Supplement: Supplementary Figure S1 — The association with concomitant drugs / lifestyle habits and alpha diversity with Shannon and Simpson index. [file crc-24-0543_supplementary_figure_s1_suppsf1.docx]

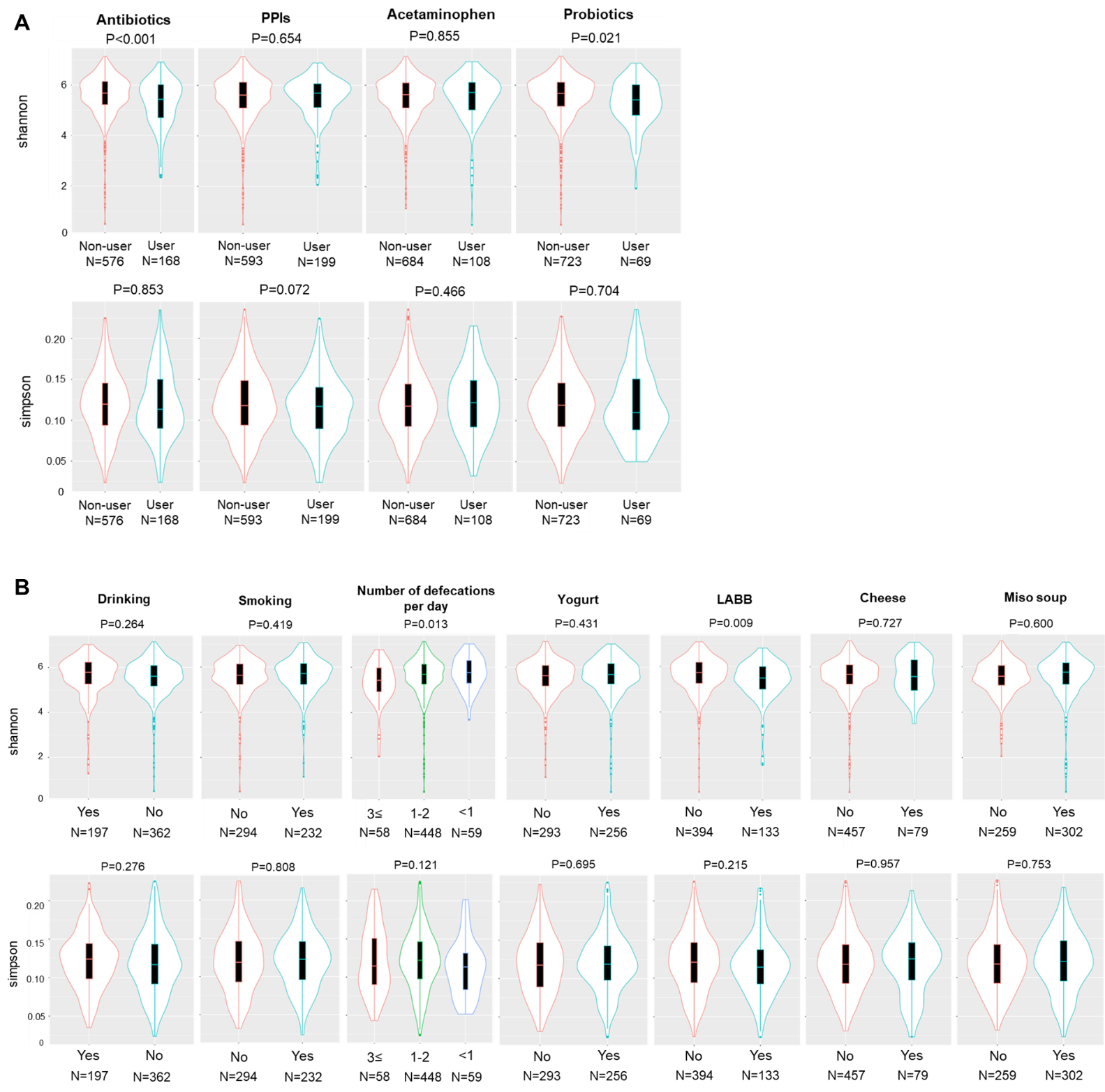


## Supplementary Figure S1: The association with concomitant drugs / lifestyle habits and alpha diversity with Shannon and Simpson index.

(A) Violin plot of alpha diversity based on Shannon and Simpson index in feces according to concomitant medication use. A two-sided P value was calculated with Welch’s t-test. (B) Violin plot of alpha diversity based on Shannon and Simpson index in feces according to lifestyle habit. A two-sided P value was calculated with Welch’s t-test. Regarding the number of defecations per day, the Kruskal-Wallis test was applied. LABB, lactic acid bacteria beverage.
