# Supplementary material for: Microbiome Landscape and Association with Response to Immune Checkpoint Inhibitors in Advanced Solid Tumors: A SCRUM-Japan MONSTAR-SCREEN Study
Source: Cancer Res Commun. 2025 May 27;5(5):857–70. doi: 10.1158/2767-9764.CRC-24-0543 (PMC12107420; doi:10.1158/2767-9764.CRC-24-0543)
Supplement: Supplementary Figure S2 — The association with concomitant drugs / lifestyle habits and beta diversity. [file crc-24-0543_supplementary_figure_s2_suppsf2.docx]

##
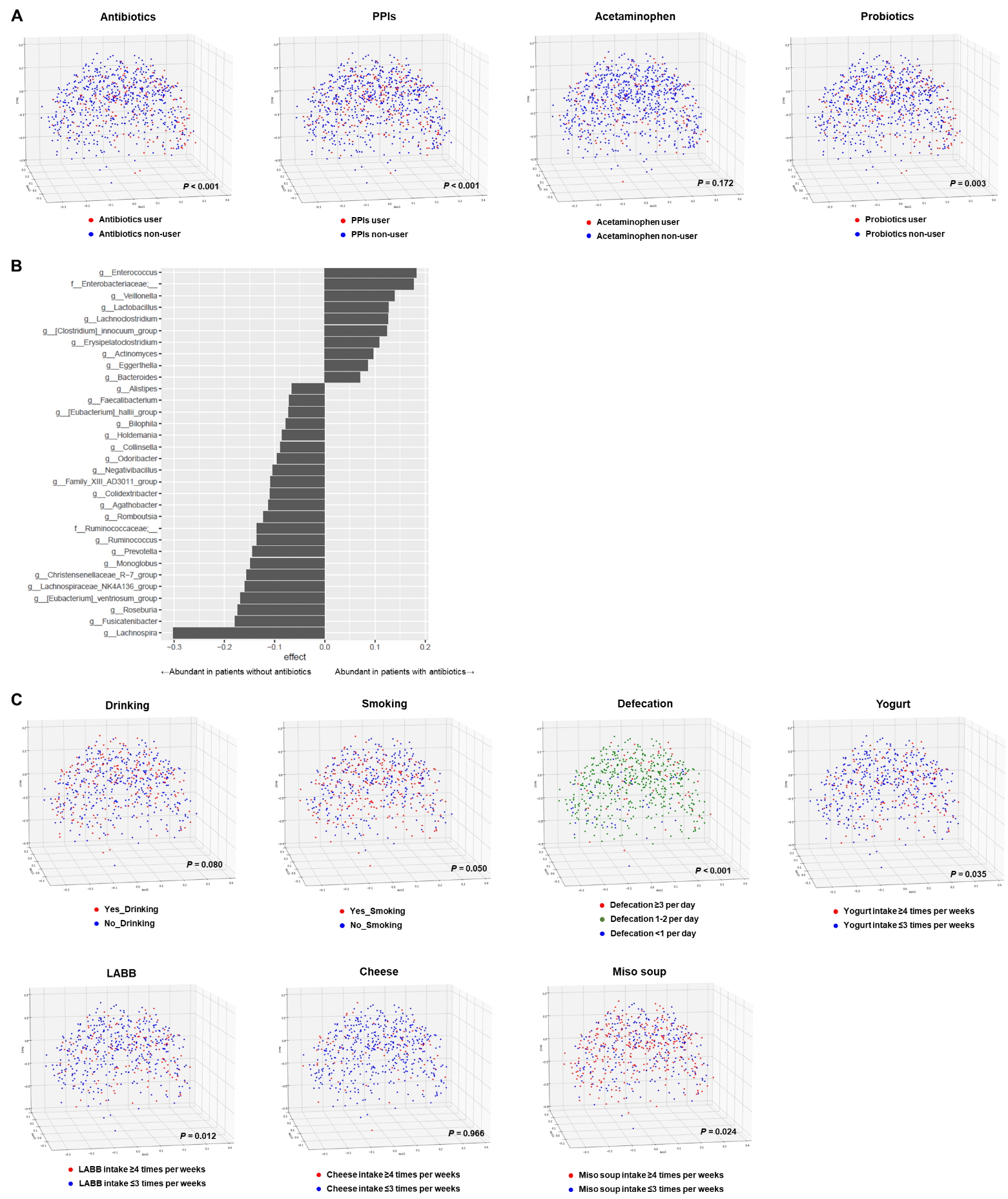
Supplementary Figure S2: The association with concomitant drugs / lifestyle habits and beta diversity.

(A) Each principal coordinate analysis presents bacterial component based on concomitant medication use or not (in all patients) and (B) The ALDEx2 analysis of flora according to with (n=168) or without (n=576) antibiotics in cohort 1. (C) Each principal coordinate analysis presents bacterial component based on lifestyle habit (in patients without antibiotics use). *P*-values of beta diversity were calculated with PERMANOVA analysis.
