# Supplementary material for: Microbiome Landscape and Association with Response to Immune Checkpoint Inhibitors in Advanced Solid Tumors: A SCRUM-Japan MONSTAR-SCREEN Study
Source: Cancer Res Commun. 2025 May 27;5(5):857–70. doi: 10.1158/2767-9764.CRC-24-0543 (PMC12107420; doi:10.1158/2767-9764.CRC-24-0543)
Supplement: Supplementary Figure S3 — The association with cancer types and alpha diversity with Shannon and Simpson index. [file crc-24-0543_supplementary_figure_s3_suppsf3.docx]

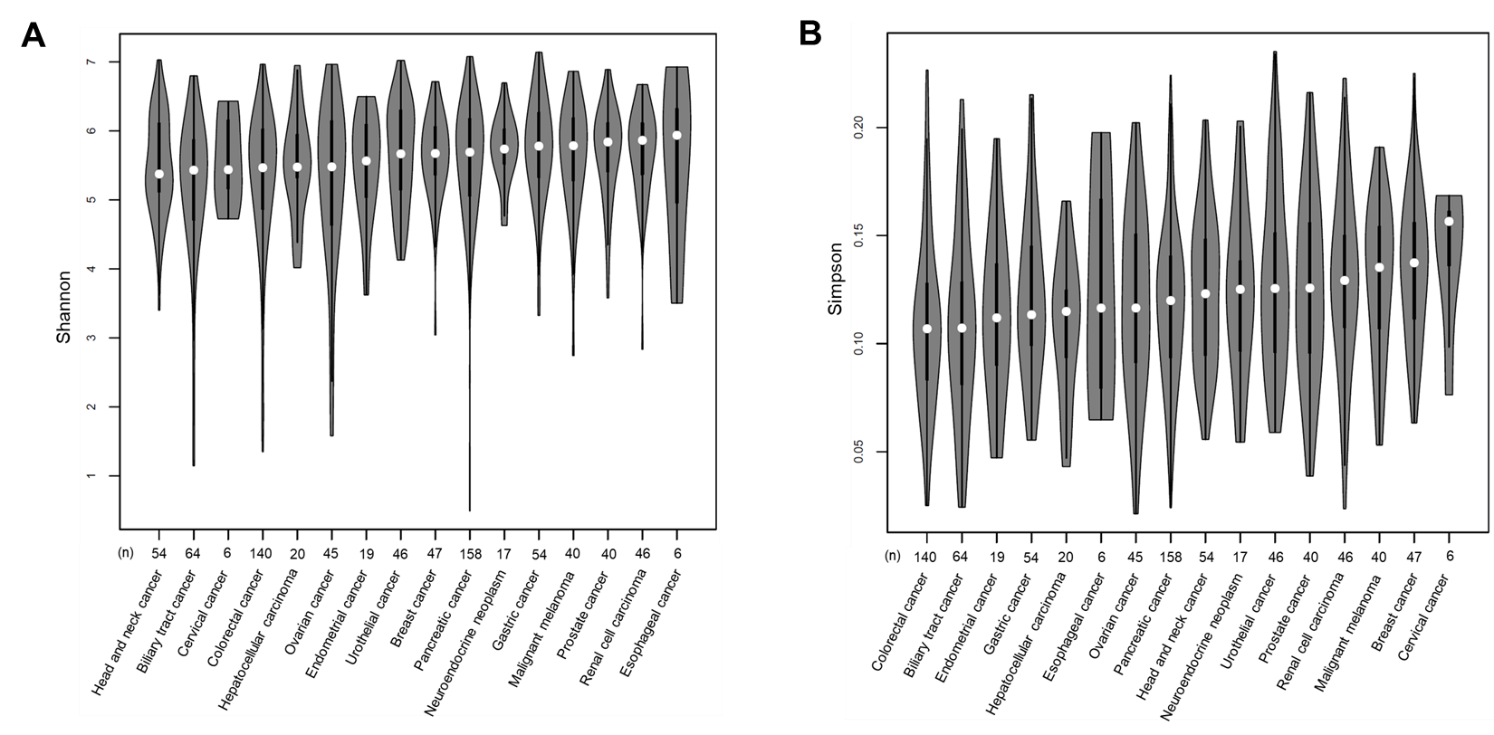


## Supplementary Figure S3: The association with cancer types and alpha diversity with Shannon and Simpson index.

Alpha diversity based on (A) Shannon index and (B) Simpson index according to cancer type, based on the analysis of at least five specimens obtained from patients in cohort 1.
