# Supplementary material for: Microbiome Landscape and Association with Response to Immune Checkpoint Inhibitors in Advanced Solid Tumors: A SCRUM-Japan MONSTAR-SCREEN Study
Source: Cancer Res Commun. 2025 May 27;5(5):857–70. doi: 10.1158/2767-9764.CRC-24-0543 (PMC12107420; doi:10.1158/2767-9764.CRC-24-0543)
Supplement: Supplementary Figure S4 — Proportion of oral bacteria in patients with upper gastrointestinal cancers feces according to the presence or absence of a primary tumor by cancer type. [file crc-24-0543_supplementary_figure_s4_suppsf4.docx]

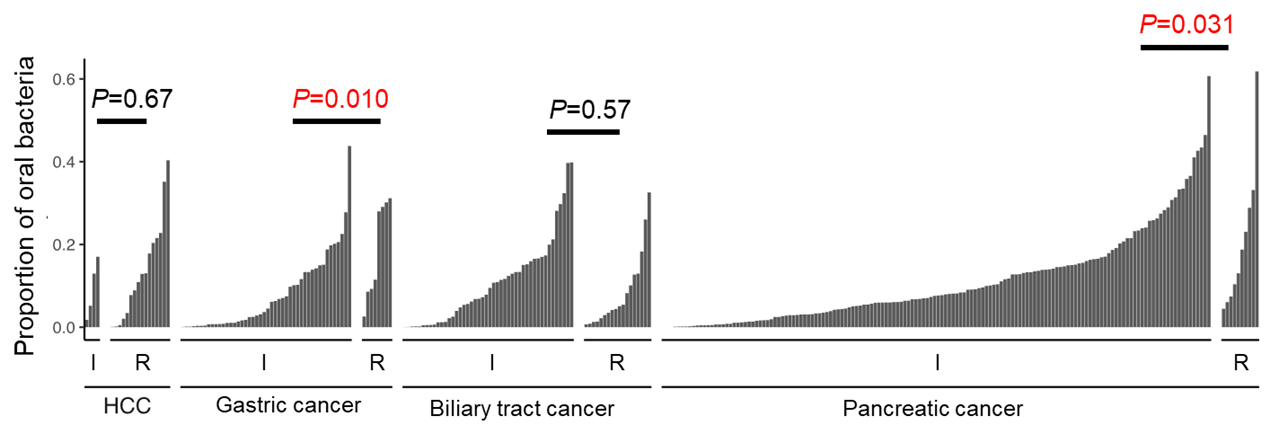


## Supplementary Figure S4: Proportion of oral bacteria in patients with upper gastrointestinal cancers feces according to the presence or absence of a primary tumor by cancer type.

HCC, hepatocellular carcinoma. I. Intact primary tumor. R: Primary tumor resection.
