# Supplementary material for: Microbiome Landscape and Association with Response to Immune Checkpoint Inhibitors in Advanced Solid Tumors: A SCRUM-Japan MONSTAR-SCREEN Study
Source: Cancer Res Commun. 2025 May 27;5(5):857–70. doi: 10.1158/2767-9764.CRC-24-0543 (PMC12107420; doi:10.1158/2767-9764.CRC-24-0543)
Supplement: Supplementary Figure S5 — Association of ICI efficacy with ASV based on treatment. [file crc-24-0543_supplementary_figure_s5_suppsf5.docx]

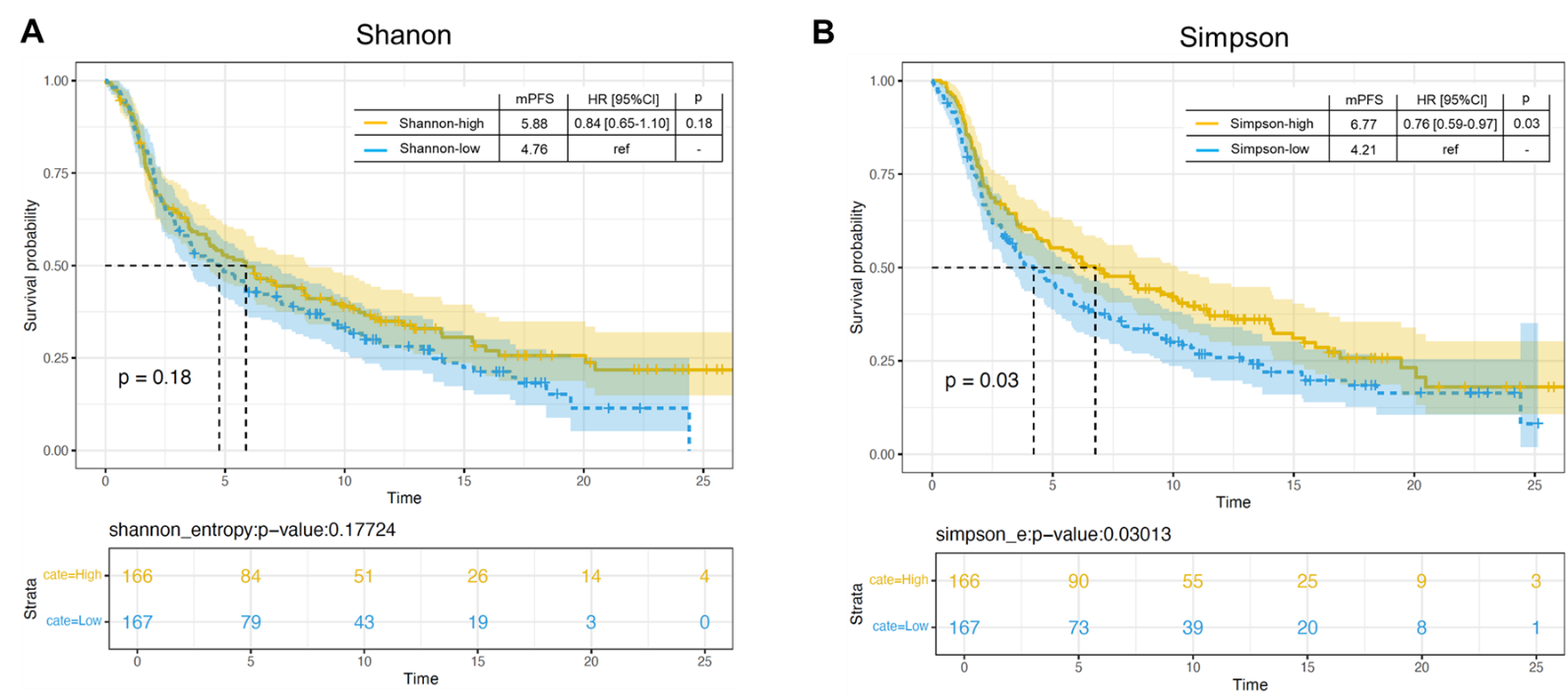


## Supplementary Figure S5: Association of ICI efficacy with ASV based on treatment.

(A) Kaplan-Meier plots of the PFS of patients treated with ICIs in high Shannon index group and low Shannon index group. The cutoff of Shannon index was median. (B) Kaplan-Meier plots of the PFS of patients treated with ICIs in high Simpson index group and low Simpson index group. The cutoff of Simpson index was median.
