# Supplementary material for: Microbiome Landscape and Association with Response to Immune Checkpoint Inhibitors in Advanced Solid Tumors: A SCRUM-Japan MONSTAR-SCREEN Study
Source: Cancer Res Commun. 2025 May 27;5(5):857–70. doi: 10.1158/2767-9764.CRC-24-0543 (PMC12107420; doi:10.1158/2767-9764.CRC-24-0543)
Supplement: Supplementary Figure S6 — Association of ICI efficacy with ASV based on treatment. [file crc-24-0543_supplementary_figure_s6_suppsf6.docx]

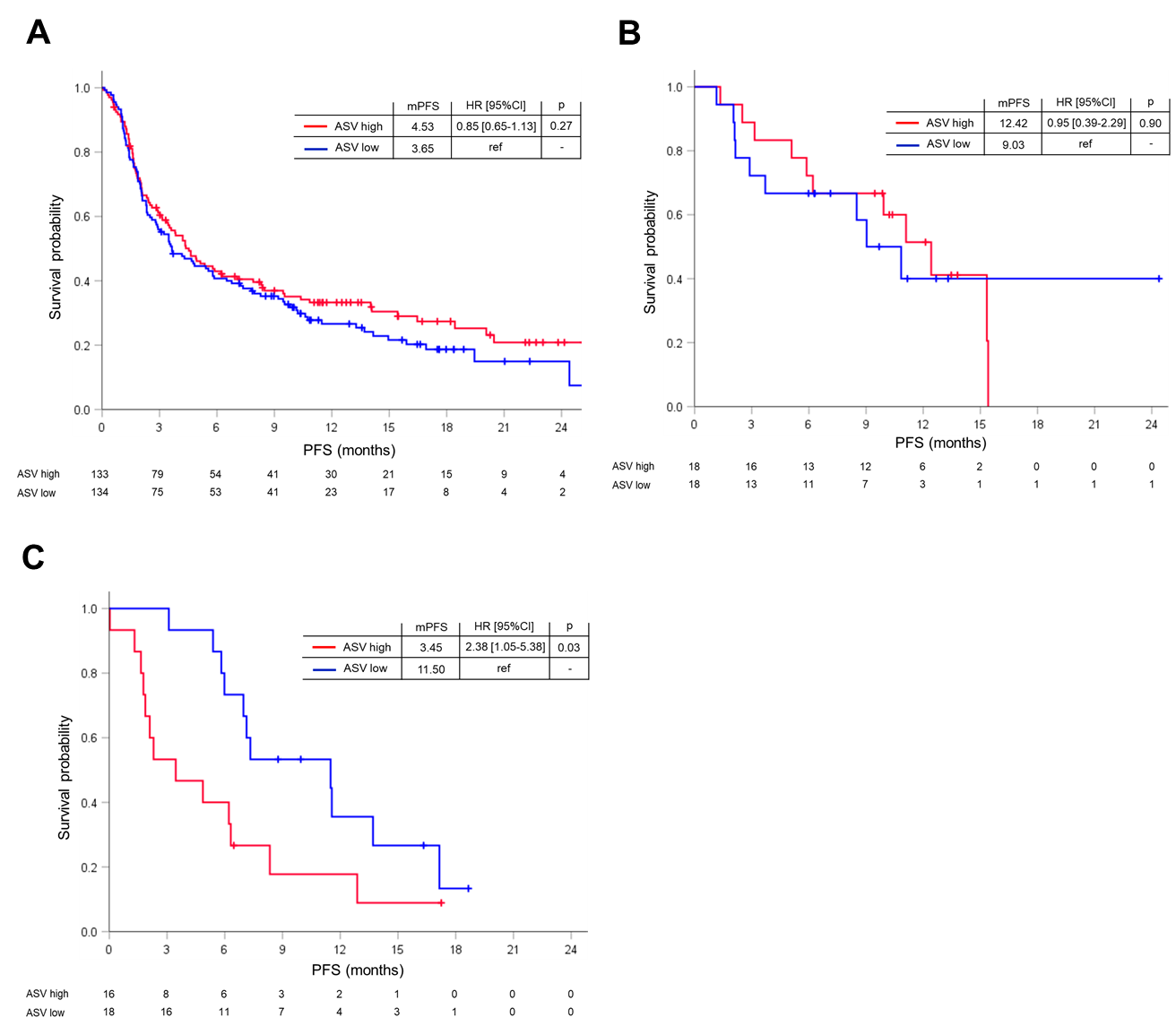


## Supplementary Figure S6: Association of ICI efficacy with ASV based on treatment.

(A) Kaplan-Meier plots of PFS in patients treated with ICIs alone based on high or low ASV count. “ICIs alone” was treated with anti-PD-1/PD-L1 monotherapy or the combination of anti-PD-1 and anti-CTLA-4 antibodies. (B) Kaplan-Meier plots of PFS in patients treated with ICIs and molecularly targeted agents based on high or low ASV count. (C) Kaplan-Meier plots of PFS in patients treated with ICIs plus chemotherapy based on high or low ASV count.
