# Supplementary material for: Microbiome Landscape and Association with Response to Immune Checkpoint Inhibitors in Advanced Solid Tumors: A SCRUM-Japan MONSTAR-SCREEN Study
Source: Cancer Res Commun. 2025 May 27;5(5):857–70. doi: 10.1158/2767-9764.CRC-24-0543 (PMC12107420; doi:10.1158/2767-9764.CRC-24-0543)
Supplement: Supplementary Figure S7 — The ALDEx2 analysis of flora according to PFS of ICI treatment. [file crc-24-0543_supplementary_figure_s7_suppsf7.docx]

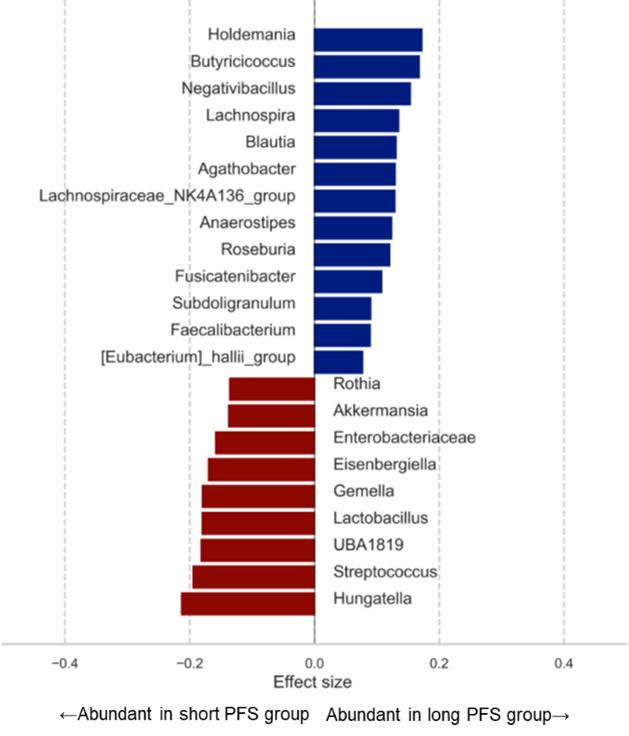


## Supplementary Figure S7: The ALDEx2 analysis of flora according to PFS of ICI treatment.

The cutoff of PFS is median. An adjusted P value < 0.05 and an absolute value of effect size > 0.2 were used to define abundant species.
