# Supplementary material for: Microbiome Landscape and Association with Response to Immune Checkpoint Inhibitors in Advanced Solid Tumors: A SCRUM-Japan MONSTAR-SCREEN Study
Source: Cancer Res Commun. 2025 May 27;5(5):857–70. doi: 10.1158/2767-9764.CRC-24-0543 (PMC12107420; doi:10.1158/2767-9764.CRC-24-0543)
Supplement: Supplementary Figure S8 — Association of ICI efficacy with concomitant medication use. [file crc-24-0543_supplementary_figure_s8_suppsf8.docx]

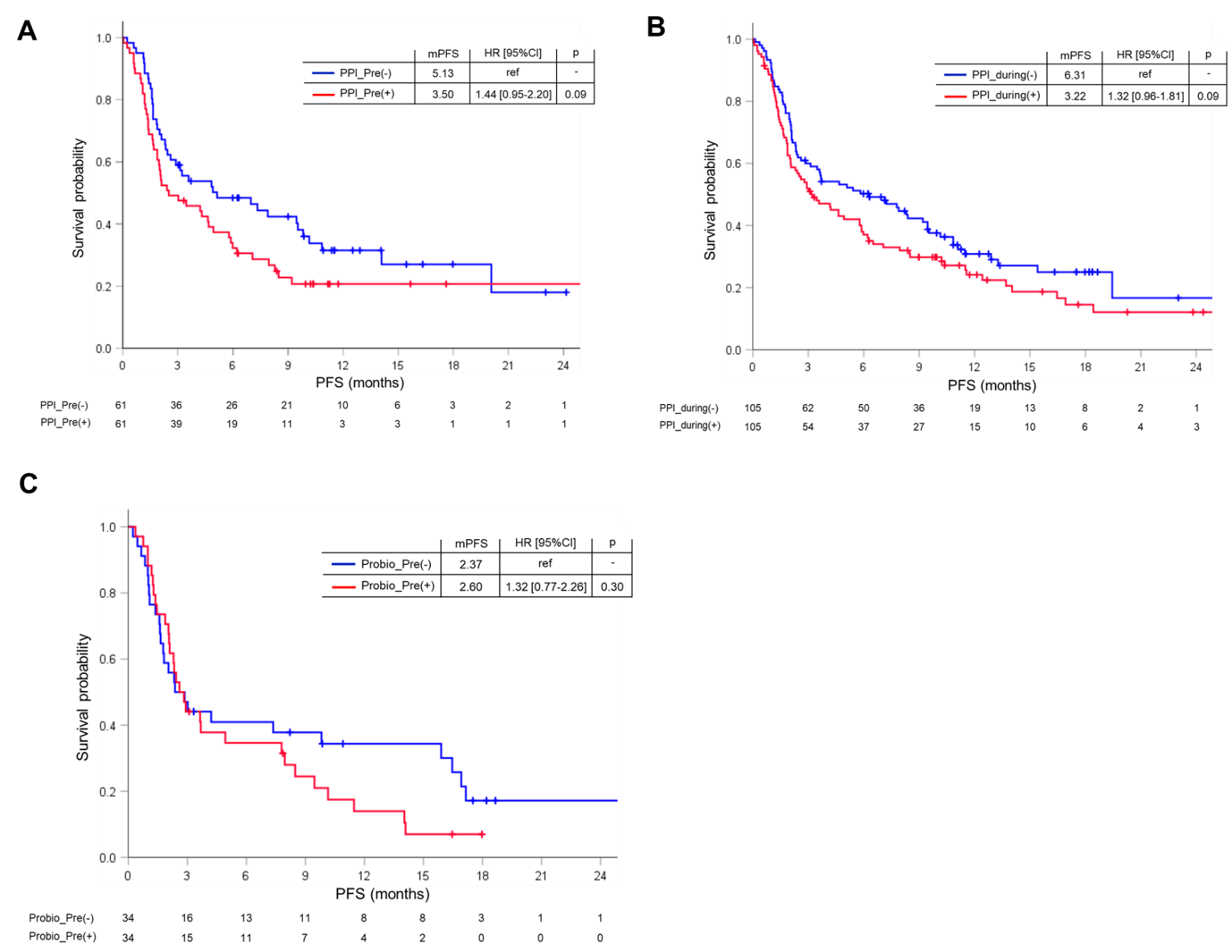


## Supplementary Figure S8: Association of ICI efficacy with concomitant medication use.

Kaplan-Meier plots of PFS (after adjusting using propensity score matching) of patients treated with ICIs based on (A) PPIs use before ICI treatment (B) PPIs use during ICI treatment (C) Probiotics use before ICI treatment.
