# Supplementary material for: Microbiome Landscape and Association with Response to Immune Checkpoint Inhibitors in Advanced Solid Tumors: A SCRUM-Japan MONSTAR-SCREEN Study
Source: Cancer Res Commun. 2025 May 27;5(5):857–70. doi: 10.1158/2767-9764.CRC-24-0543 (PMC12107420; doi:10.1158/2767-9764.CRC-24-0543)
Supplement: Supplementary Figure S9 — ALDEx2 analysis of the comparison of the fecal microbiome in patients with or without concomitant medication use before ICI treatment. [file crc-24-0543_supplementary_figure_s9_suppsf9.docx]

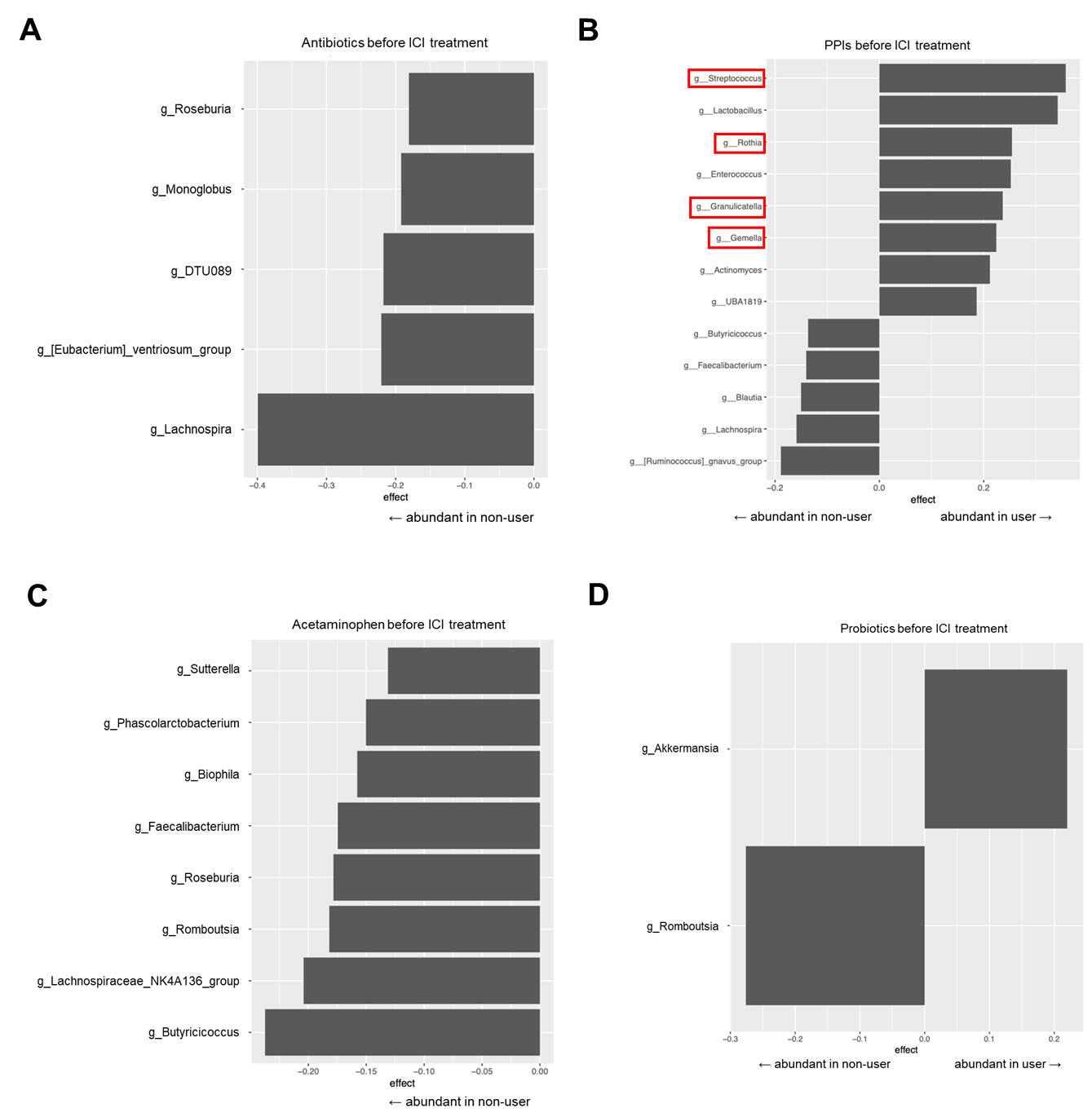


## Supplementary Figure S9: ALDEx2 analysis of the comparison of the fecal microbiome in patients with or without concomitant medication use before ICI treatment.

(A) Antibiotic users (n=57) and non-users (n=276); (B) PPI users (n=61) and non-users (n=272); (C) acetaminophen users (n=48) and non-users (n=285); (D) probiotic users (n=34) and non-users (n=299). An adjusted P value < 0.05 and an absolute value of effect size > 0.2 were used to define abundant species. Species defined as oral bacteria are indicated by red boxes.
