# Supplementary material for: Microbiome Landscape and Association with Response to Immune Checkpoint Inhibitors in Advanced Solid Tumors: A SCRUM-Japan MONSTAR-SCREEN Study
Source: Cancer Res Commun. 2025 May 27;5(5):857–70. doi: 10.1158/2767-9764.CRC-24-0543 (PMC12107420; doi:10.1158/2767-9764.CRC-24-0543)
Supplement: Supplementary Figure S10 — ICI efficacy between two categories of the proportion of oral bacteria in feces. [file crc-24-0543_supplementary_figure_s10_suppsf10.docx]

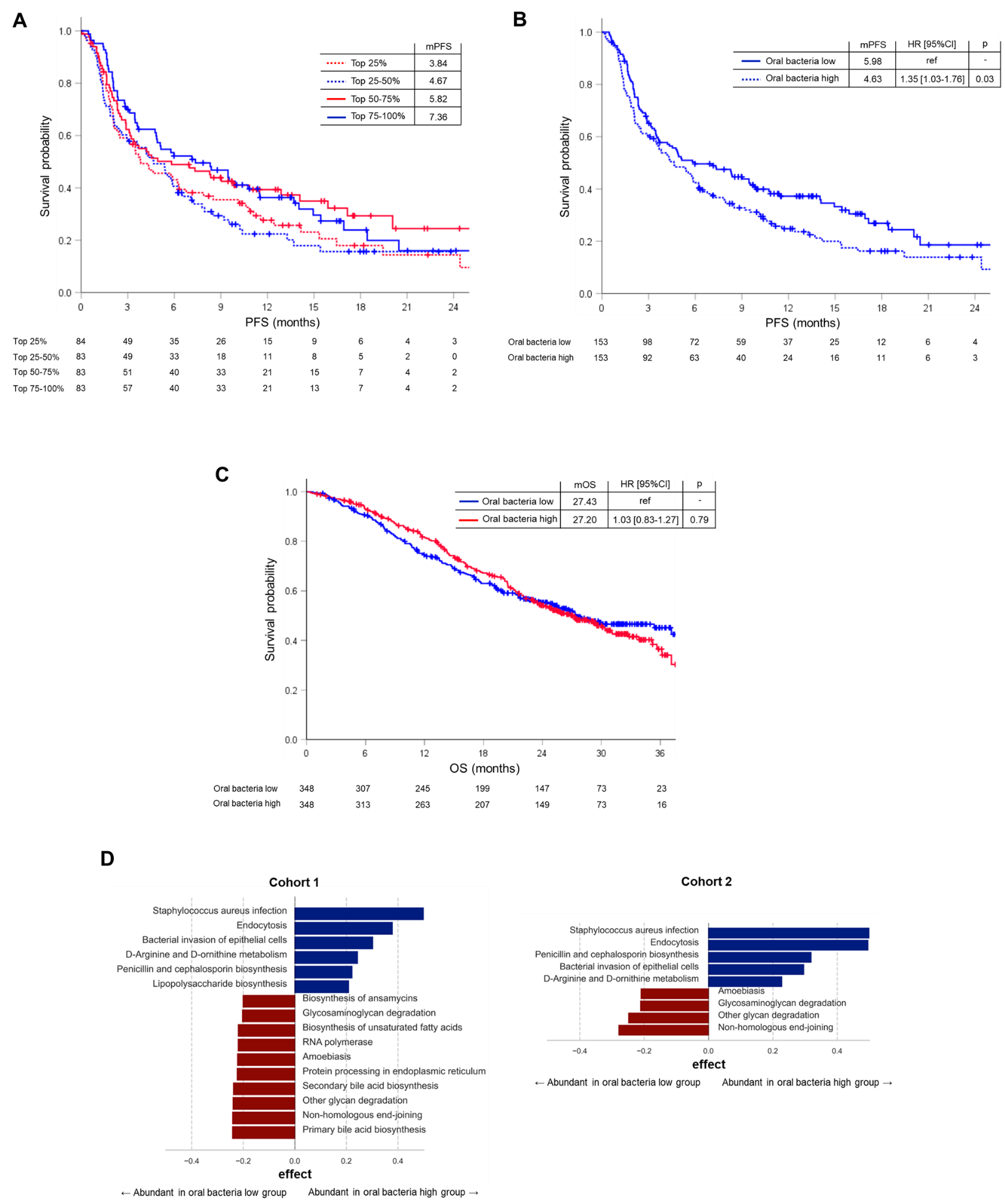


## Supplementary Figure S10: ICI efficacy between two categories of the proportion of oral bacteria in feces.

(A) Kaplan-Meier plots of PFS of patients treated with ICIs based on the proportion of oral bacteria in feces. Patients were divided into four groups by the proportion of oral bacteria. (B) Kaplan-Meier plots of PFS of patients treated with ICIs based on the proportion of oral bacteria in feces, after adjustment with propensity score matching. “Oral bacteria high” was defined as patients with the proportion of oral bacteria above median. (C) Kaplan-Meier plots of OS according to the proportion of oral bacteria in patients of cohort 1. (D) The ALDEx2 analysis of pathway in Cohort 1 and 2 according to proportion of oral bacteria in feces.
