# Supplementary material for: Microbiome Landscape and Association with Response to Immune Checkpoint Inhibitors in Advanced Solid Tumors: A SCRUM-Japan MONSTAR-SCREEN Study
Source: Cancer Res Commun. 2025 May 27;5(5):857–70. doi: 10.1158/2767-9764.CRC-24-0543 (PMC12107420; doi:10.1158/2767-9764.CRC-24-0543)
Supplement: Supplementary Figure S11 — Validation with publicly data about oral bacteria and the efficacy of ICIs [file crc-24-0543_supplementary_figure_s11_suppsf11.docx]

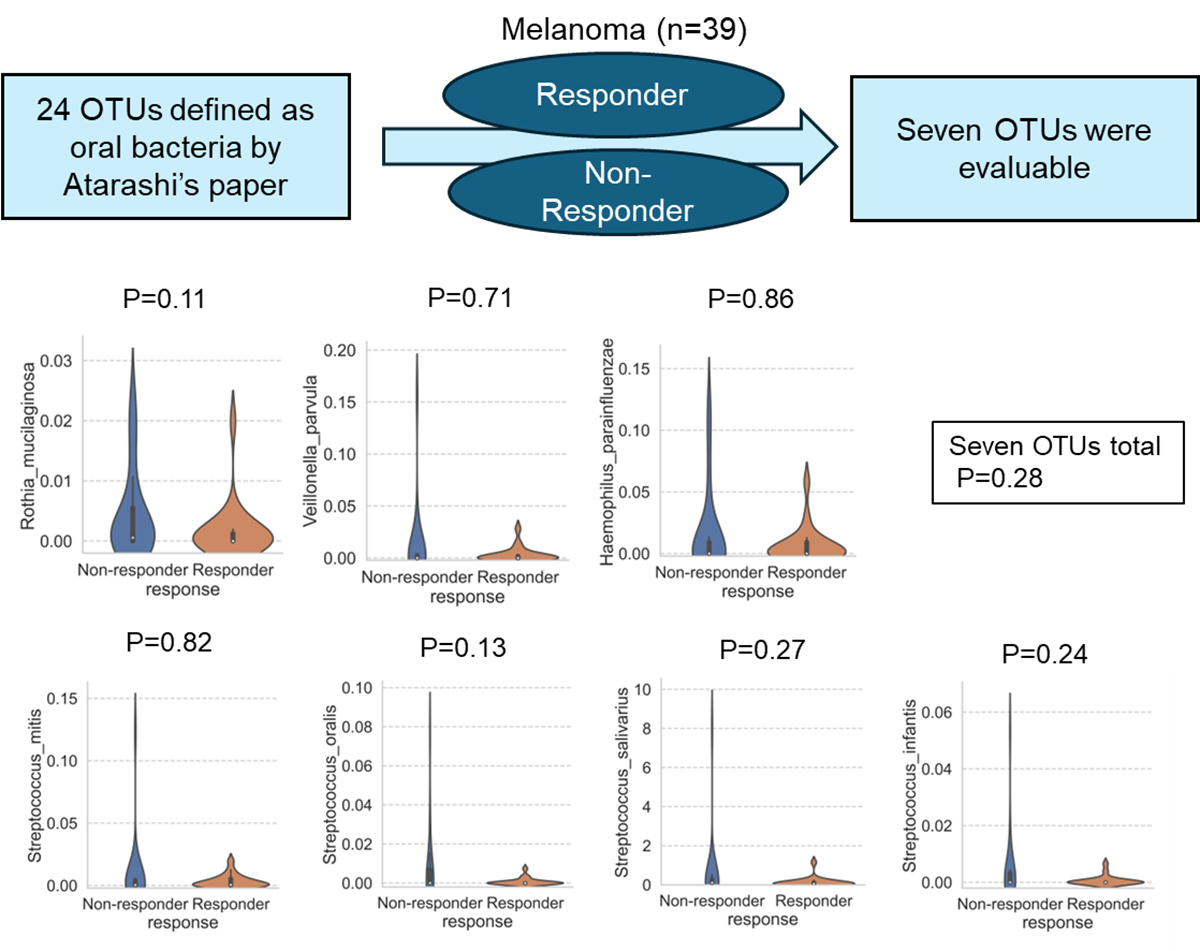


## Supplementary Figure S11: Validation with publicly data about oral bacteria and the efficacy of ICIs.

Violin plot of proportion of oral bacteria and ICIs efficacy using public dataset of 39 patients with malignant melanoma treated with ICIs. Seven of 24 OTUs we defined “oral bacteria” were evaluable in this dataset.
