# Supplementary figures and images for: Microbiome Landscape and Association with Response to Immune Checkpoint Inhibitors in Advanced Solid Tumors: A SCRUM-Japan MONSTAR-SCREEN Study
Source: Cancer Res Commun. 2025 May 27;5(5):857–70. doi: 10.1158/2767-9764.CRC-24-0543 (PMC12107420; doi:10.1158/2767-9764.CRC-24-0543)

## Supplementary Table S1: Patient characteristics of PPI user and non-user in Cohort 1


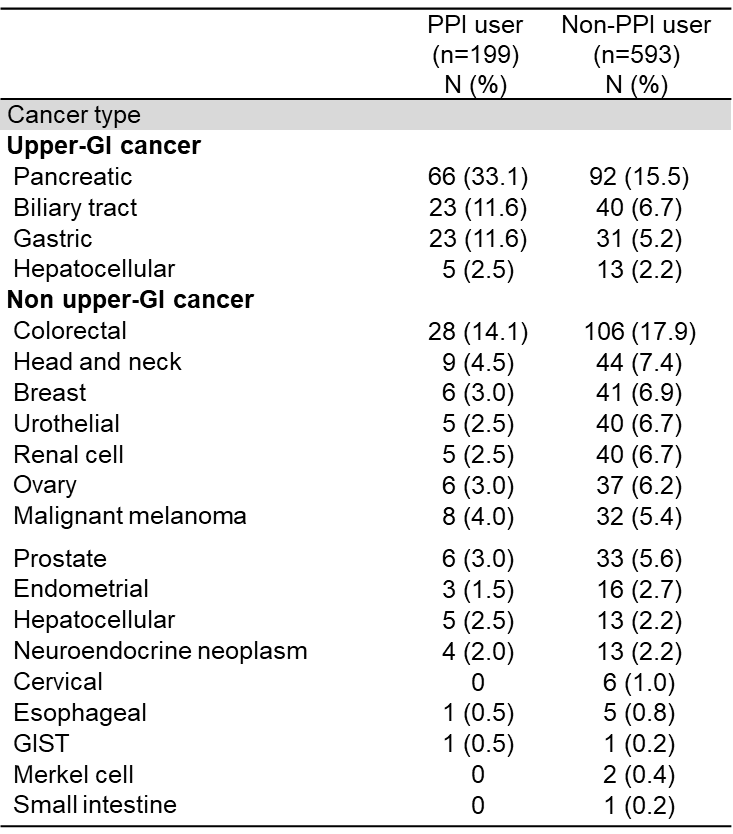

Supplement: Supplementary Table S1 — Patient characteristics of PPI user and non-user in Cohort 1. [file crc-24-0543_supplementary_table_s1_suppst1.docx]

## Supplementary Table S2: Concomitant drug usage in each cancer type in Cohort 2


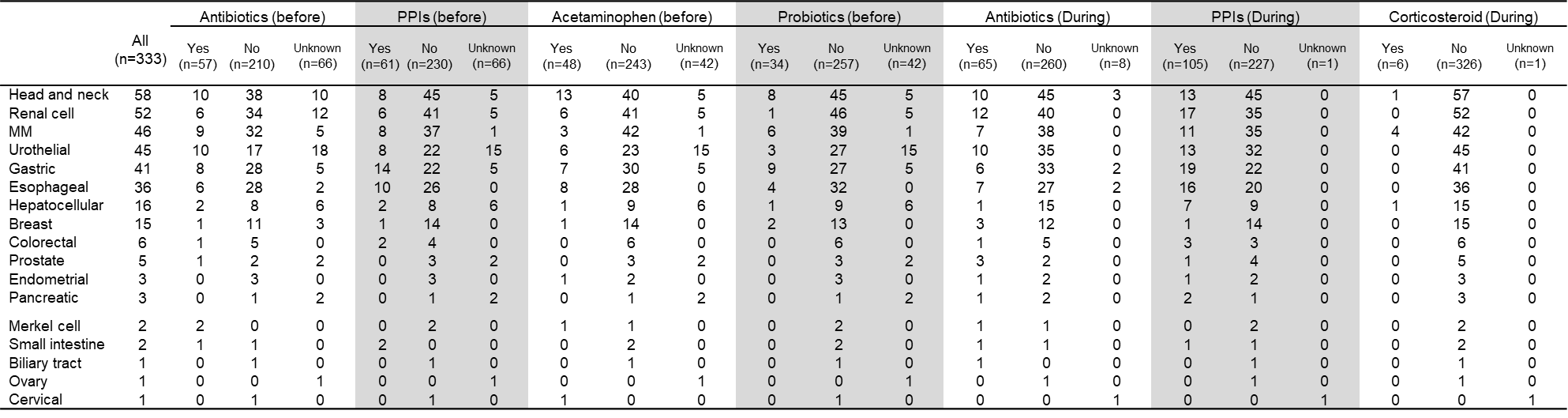

Supplement: Supplementary Table S2 — Concomitant drug usage in each cancer type in Cohort 2. [file crc-24-0543_supplementary_table_s2_suppst2.docx]

## Supplementary Table S4: Multivariate analysis in PFS of ICIs in cohort 2


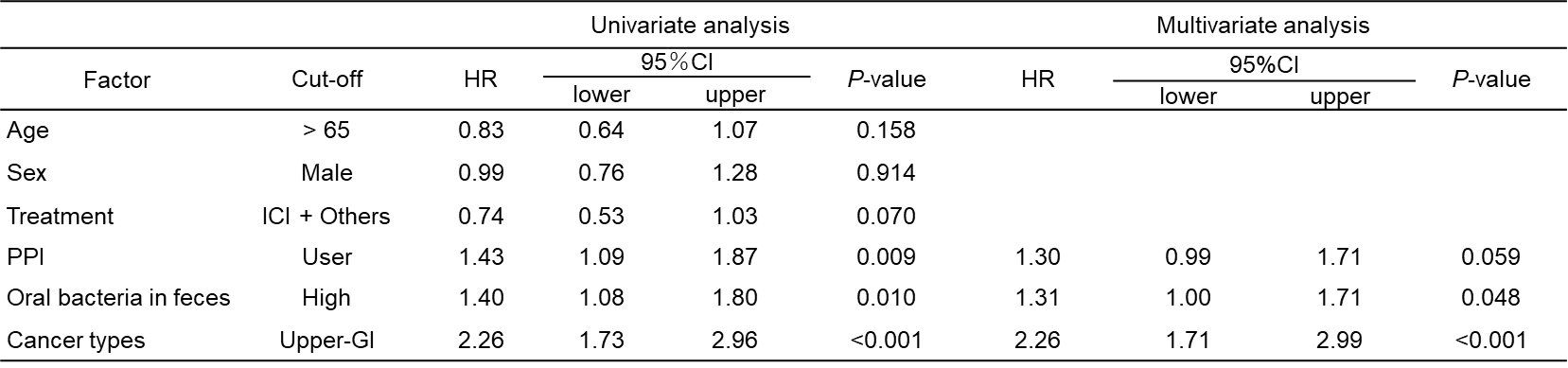

Supplement: Supplementary Table S4 — Multivariate analysis in PFS of ICIs in cohort 2. [file crc-24-0543_supplementary_table_s4_suppst4.docx]
