# Supplementary material for: Microbiome Landscape and Association with Response to Immune Checkpoint Inhibitors in Advanced Solid Tumors: A SCRUM-Japan MONSTAR-SCREEN Study
Source: Cancer Res Commun. 2025 May 27;5(5):857–70. doi: 10.1158/2767-9764.CRC-24-0543 (PMC12107420; doi:10.1158/2767-9764.CRC-24-0543)

## Supplementary Table S3: Patient characteristics of two groups based on the proportion of oral bacteria in feces in cohort 2


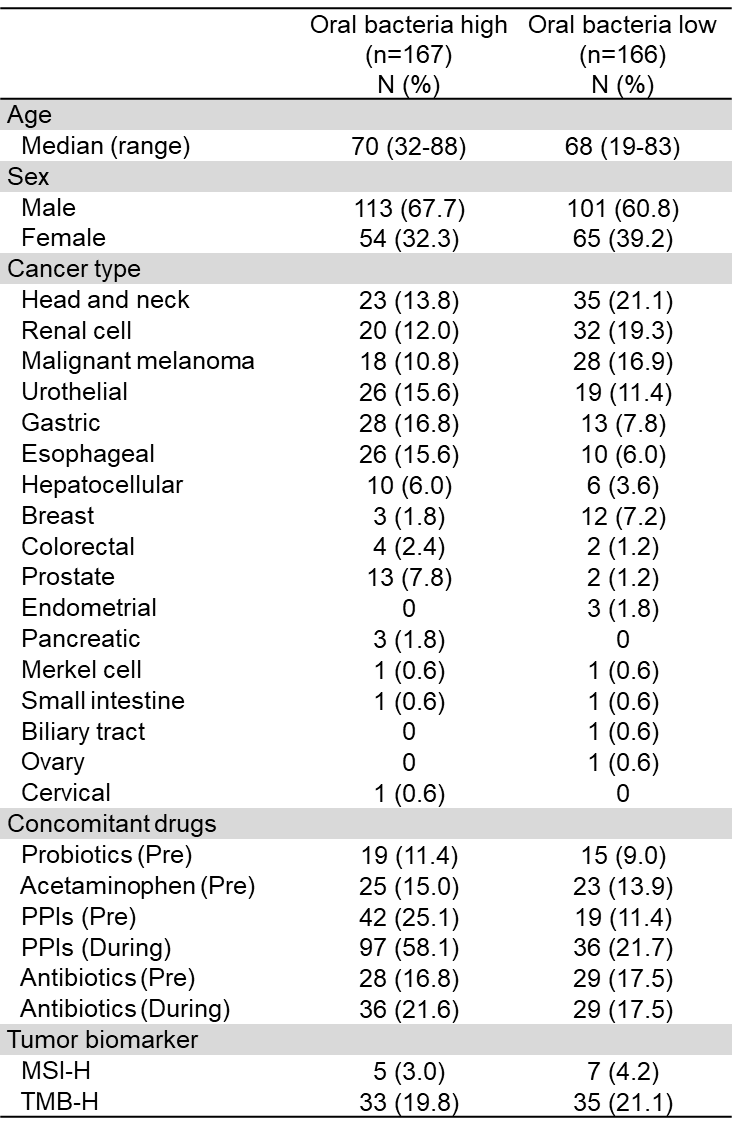

Supplement: Supplementary Table S3 — Patient characteristics of two groups based on the proportion of oral bacteria in feces in cohort 2. [file crc-24-0543_supplementary_table_s3_suppst3.docx]
